# Large‐Scale In Situ Formation Perovskite Quantum Dots/Elastomer Composite for High‐Performance White Light‐Emitting Diodes

**DOI:** 10.1002/advs.202522934

**Published:** 2026-01-27

**Authors:** Yuxian Su, Shirong Yu, Hao Shen, Dongdong Kang, Beibei Wang, Xuebin Yu, Yongyin Kang

**Affiliations:** ^1^ Key Laboratory of Extreme Environment Functional Materials Yiwu Research Institute of Fudan University Yiwu China; ^2^ Department of Materials Science Fudan University Shanghai China

**Keywords:** flexible PQDs devices, halogenated butyl rubber, in situ formation, large‐scale, stability

## Abstract

Metal halide perovskite quantum dots (PQDs) are promising emitters for light conversion but suffer from poor compatibility and scalability in polymer composites. Herein, we report a one‐step, solvent‐free mechanochemical strategy for the in situ synthesis of CsPbX_3_ (Br/Cl) QDs within halogenated butyl rubber (HIIR) via open‐mill shear, yielding monodisperse 2.6 nm CsPbBr_3_ QDs uniformly embedded in a topological entanglement network. The CsPbX_3_/HIIR composite exhibited a narrow emission at 515 nm (FWHM 17 nm), photoluminescence quantum yield of 91%, and ultra‐long lifetime of 1189 ns. Spectroscopic and mechanistic studies revealed that the in situ‐generated IIR‐OOCC_17_H_35_ chains create an interfacial allyl ester passivation layer that suppresses surface traps and blocks H_2_O/O_2_ ingress. Consequently, films retained > 90% initial intensity after 30‐day ambient storage, 99.8% after 30‐day water immersion, and 44% after 500 h blue‐light irradiation (5000 nit). Flexible white LEDs achieved standard white emission (0.333, 0.338), color‐rendering index of 55.7, and wide color gamut of 132% NTSC. The low‐cost and high‐efficiency manufacturing process can be readily scaled to 17 × 17 cm^2^ films, offering an industrially viable route for stretchable displays, X‐ray scintillators, and wearable photonics.

## Graphical abstract

A solvent‐free, open‐mill shear protocol nucleates 2.6 nm CsPbBr_3_ QDs uniformly within brominated butyl rubber. The emitters showed 91% PLQY, 1189 ns lifetime, and > 90% retention after 30‐day ambient storage and water immersion, respectively. Board‐to‐board forms 17 × 17 cm^2^ flexible films for 132% NTSC white LEDs, scintillators, and stretchable displays.

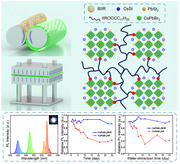

## 1 Introduction

Metal halide perovskite quantum dots (PQDs) possess exceptional optoelectronic properties such as high light absorption coefficients, narrow full width at half maximum (FWHM), wide color gamut, tunable emission wavelengths, high luminescence efficiency, and excellent solution processability, granting them broad application potential in light conversion fields like lighting, displays, lasers, concentrators, and scintillators [1, 2, 3, 4, 5, 6, 7]. To date, perovskite light‐emitting diodes (PeLEDs) fabricated on rigid glass substrates have achieved external quantum efficiencies (EQEs) exceeding 20% in both red (25.8% for CsPbI_3_‐based devices) [8] and green (28.1% for CsPbBr_3_‐based devices) [9] spectral regions, approaching the threshold for commercial applications. However, the performance of flexible perovskite devices still lags significantly behind that of rigid devices, primarily due to two critical challenges. First, the significant modulus mismatch between the flexible substrates and the perovskite layer induces stress concentration at the interface under bending deformation, leading to the formation of microcracks and structural defects that degrade the device performance [10]. Second, the intrinsic ionic nature of perovskite materials makes them highly susceptible to moisture and oxygen, resulting in exponentially accelerated device degradation and shortened operational lifetime under ambient conditions [11]. Therefore, the development of the perovskite material that combines high mechanical flexibility with excellent environmental stability has become a crucial research direction for advancing flexible optoelectronics.

Embedding PQDs into a polymer matrix has been demonstrated to be an effective physical stabilization strategy. The long chains of polymers can form dense encapsulation structures that effectively block the ingress of moisture and oxygen, significantly enhancing the environmental stability of the QDs and improving their processability [12, 13, 14, 15, 16]. Furthermore, such encapsulation markedly suppresses lead ion leaching, mitigating a key environmental concern associated with perovskite materials in practical applications [17]. Currently, strategies for preparing PQD‐polymer composites are broadly classified into two categories: i) pre‐synthesizing QDs followed by blending with polymers; and ii) in situ formation of QDs within the polymer matrix. The former approach involves multiple steps—QD synthesis, purification, and mixing—making the process cumbersome [16]. Moreover, during subsequent processing steps such as spin‐coating or thermal treatment, the QDs are prone to degradation due to ligand desorption or environmental attack, and their high surface energy often causes aggregation, leading to diminished luminescence performance. In contrast, the in situ preparation method involves blending precursors with the polymer, followed by thermal or mechanical induction to directly form the QDs within the polymer matrix. This process allows polymer chains to effectively anchor nucleation sites and passivate surface defects, simultaneously achieving uniform nanoscale dispersion and enhancing the intrinsic stability of the QDs [4]. Nevertheless, owing to the insufficiently precise control over the in situ nucleation and growth kinetics, QDs produced by this method often exhibit a broad size distribution and relatively low photoluminescence quantum yield (PLQY), which to some extent limits their practical application [18].

Commonly used flexible matrix materials, such as polyethylene terephthalate (PET), polyethylene naphthalate (PEN), polystyrene (PS), polymethyl methacrylate (PMMA), polydimethylsiloxane (PDMS), and polyimide (PI), have been proven to enhance the chemical stability of perovskite QDs and offer certain barrier properties [19, 20, 21, 22]. However, they possess inherent mechanical limitations, such as high modulus, significant brittleness, and insufficient flexibility and fatigue durability, making it difficult to maintain structural integrity under repeated bending or stretching with large deformation, thereby restricting their long‐term application in flexible display devices. Rubber elastomers exhibit unique advantages in the next‐generation flexible display technology owing to their high elasticity, reversible deformability, and good biocompatibility [23, 24, 25, 26]. Their low modulus characteristics help dissipate stress during deformation, providing an ideal platform for constructing wearable, foldable, and even stretchable optoelectronic devices. Among them, brominated butyl rubber (BIIR) exhibits the best barrier properties among common rubbers, attributed to the highly regular symmetric methyl group arrangement along its isoprene‐derived molecular chains, which results in an extremely small free volume [27, 28]. However, integrating PQDs efficiently and stably into the BIIR matrix still faces two major challenges: first, controlling the compatibility and dispersion homogeneity at the QD‐rubber interface; and second, achieving firm embedding and long‐term stability of the QDs within the elastic network while maintaining their high luminescence performance. Systematically addressing these challenges is critical for advancing the practical application of PQD‐rubber composites in flexible display technology.

Here, based on the halogenated butyl rubber with excellent barrier properties, flexibility, and plasticity as the halogen precursor and polymer substrate, we have developed a one‐step method to fabricate high‐stability flexible perovskite devices. This strategy is highly versatile, low‐cost, and can be scaled up for commercial production. Specifically, stearic acid cesium (CsSt) and stearic acid lead (PbSt_2_) were selected as the metal precursors because of their hydrocarbon chains with good polymer compatibility. Under the shear action of the roll mill, these precursors readily form highly efficient luminescent perovskite quantum dots in situ, which were uniformly dispersed in the polymer. Unlike the PQD/polymer materials produced using traditional wet chemical methods, this strategy is solvent‐free throughout the process. The synthesized composite materials exhibited excellent stability in storage, light, heat, and water environments. Moreover, the composite materials have good flexibility, and the product shape can be easily controlled. The synthesis method is simple, low‐cost, and environmentally friendly, and has the potential for large‐scale industrial applications in flexible display devices.

## 2 Results and Discussion

### 2.1 Design for the In Situ Formation of Ultra‐Flexible PQDs Light Conversion Films

**FIGURE 1:**
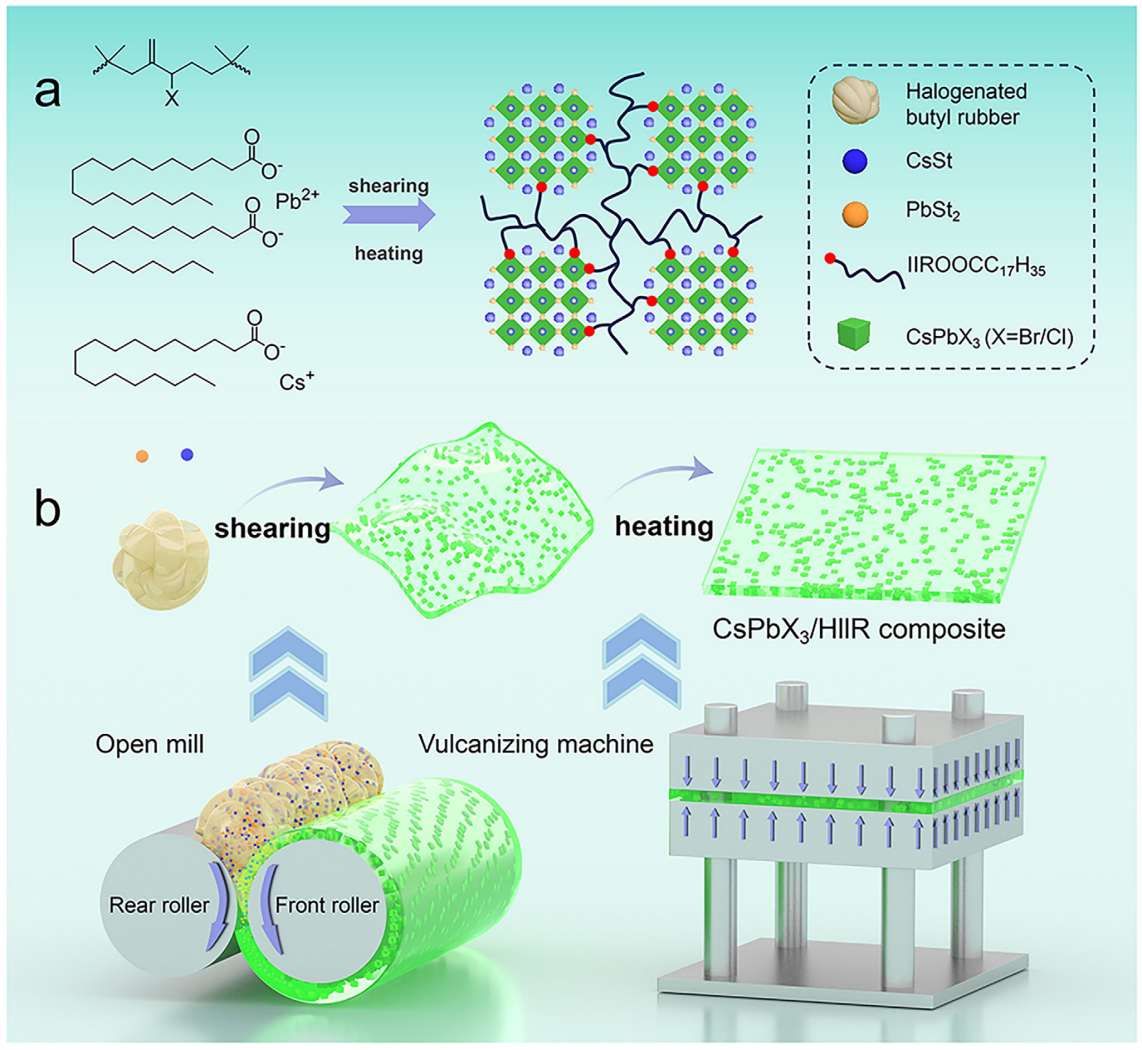
(a) Schematic diagram of the reaction structure from the precursor to the CsPbX_3_/HIIR composite film. (b) Synthesis process diagram of CsPbX_3_/HIIR composite film.

The in situ formation of perovskite quantum dots (PQDs) was conducted using an open mill with a halogenated butyl rubber as the reaction environment, with CsSt and PbSt_2_ added as cesium and lead sources, respectively. The key point is that the halogenated butyl rubber simultaneously served as the X^−^ (Cl/Br) source and the polymer‐inert protective layer. The symmetrical methyl arrangement in the isoprene segments of halogenated butyl rubber results in tightly packed molecular chains and an extremely small free volume, providing the optimal barrier properties among the general rubber products. This effectively blocks the inward penetration of environmental water and oxygen molecules, preventing contact with the perovskite QDs. Moreover, the halogen atom directly attached to the carbon adjacent to the double bond (allylic position) possesses extremely high reactivity, and the C─X bond readily breaks under the action of catalysts, shear, heat, etc., reacting with the cesium and lead sources to form the perovskite QDs. As shown in Figure 1, the core components of the open mill consisted of two rollers with a width of 350 mm and a diameter of 160 mm, maintaining a roller speed ratio of 1:1.35. The roller surface temperature was maintained (25°C) through cooling water circulation. First, the halogenated butyl rubber was introduced between the front and rear rollers. Owing to the friction generated by the roller rotation, the rubber was drawn into the gap, and the confined gap compressed the rubber, causing initial softening and adhesion to the roller surface, forming a continuous layer. Subsequently, the cesium stearate and stearic acid lead precursors were introduced between the two rollers. Through the speed ratio difference between the front and rear rollers (fixed at 1:1.35), the polymer molecular chains were stretched and broken, and the ionic reaction and uniform mixing of CsPbX_3_/HIIR composite were completed simultaneously under the action of shearing force (analogous to a “dough‐kneading” principle). Under the mechanical shearing force, the precursor salts dissociated into Cs^+^ and Pb^2+^ ions. The C─Br bond in the halogenated butyl rubber broke under the action of the catalysts (Cs^+^, Lewis acid Pb^2+^), forming Br^−^ ions. Subsequently, driven by their relatively low formation energy, these ions recombined through ionic bonds to form the CsPbX_3_ quantum dots.

To form a smooth QD‐rubber composite film, the rubber compound was further heated and pressurized using a plate vulcanizing machine. The molecular chains undergo rearrangement and flow through rapid segmental movements, thus uniformly filling the entire space of the mold. After a certain period of time for shaping, a CsPbX_3_/HIIR composite film with a uniform thickness was obtained. This process enabled the in situ formation of perovskite quantum dots within the polymer matrix. The entire synthesis process requires no organic solvents, is operationally simple, low‐cost, and suitable for large‐scale manufacturing, facilitating the transition of perovskite quantum dot production from laboratory research to industrial engineering.

### 2.2 Ultra‐Flexible PQDs Light Conversion Films Performances

**FIGURE 2:**
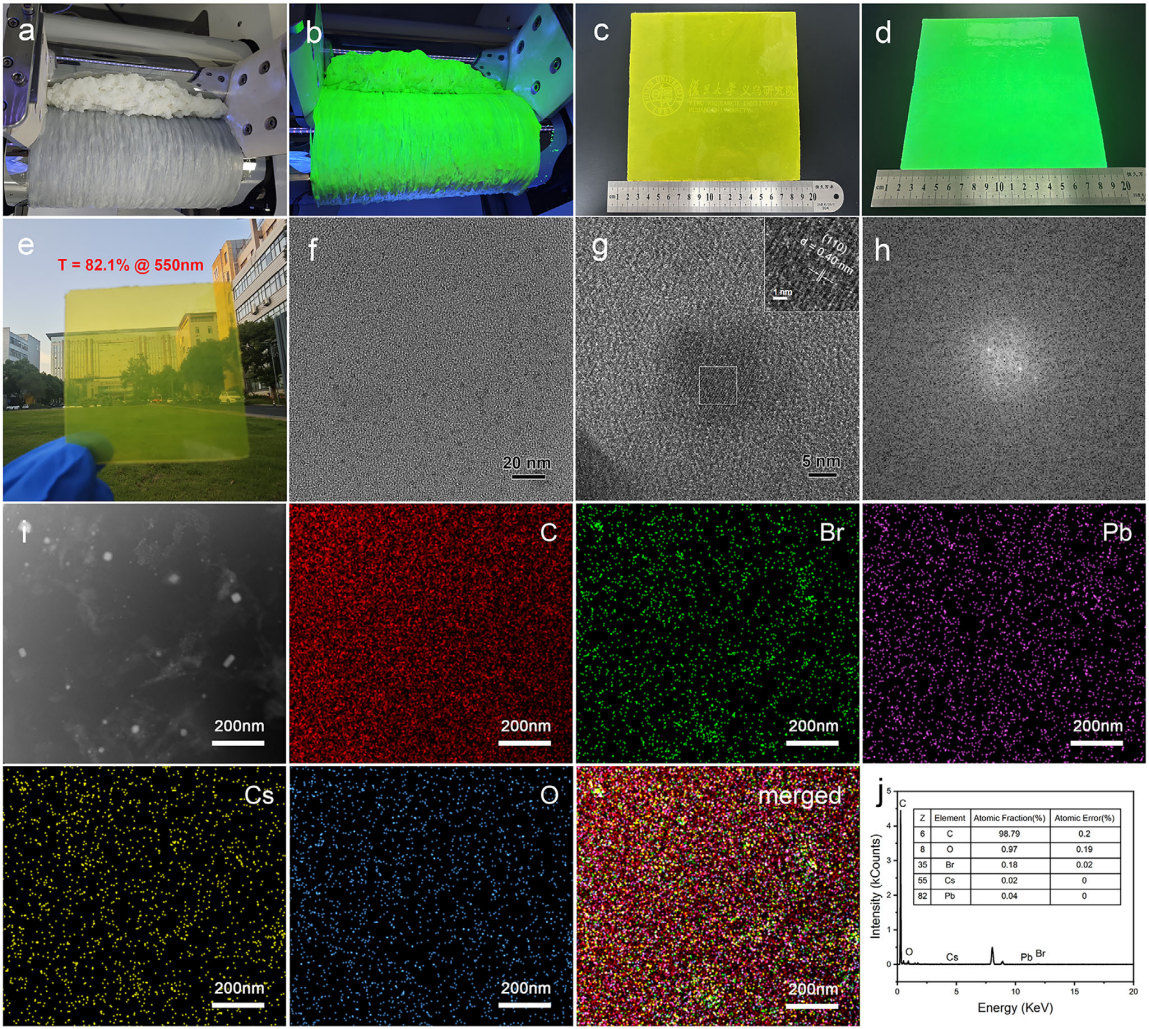
(a) Digital images of pure BIIR and (b) CsPbBr_3_/BIIR composite fabricated by an open mill. (c) Digital image of CsPbBr_3_/BIIR composite film with a large area fabricated by the hot‐pressing technique and (d) corresponding digital image of the film under UV light irradiation. (e) Digital images of CsPbBr_3_/BIIR composite film with the transmittance 82.1% @ 550 nm. (f) TEM image of CsPbBr_3_/BIIR composite film. (g) Enlarged TEM image and corresponding FFT pattern (h) of CsPbBr_3_/BIIR composite film. (i) Elemental mapping spectra and (j) EDS analysis of the CsPbBr_3_/BIIR composite film.

To fabricate large‐area flexible quantum dot light conversion films, we selected the halogenated butyl rubber as the halogen precursor and polymer matrix, respectively. Because Since perovskite quantum dots have a halogen‐dependent band gap, to deeply investigate the halogen dependence of CsPbBr_3_ perovskite nanocrystals, the amounts of lead and cesium sources were varied while keeping the amount of the halogen source (halogenated butyl rubber) constant, to study the effect of Cs, Pb, and Br stoichiometry on the optical properties of the quantum dots. Specifically, under the condition of a Cs:Pb molar ratio of 1:1, the molar ratios of Cs, Pb, and Br were regulated by changing the feeding amounts of lead and cesium salts. The mixture was then mixed for 10 min using a roll mill at a roller speed ratio of 1:1.35, as detailed in the Supplementary Material 1.1. Under bromide‐rich conditions, a higher Br content in the reaction results in the production of more [PbBr_6_]^4−^ octahedral domains. When cesium ions diffuse into the interstitial voids, the CsPbBr_3_ nuclei spontaneously form. During the nucleation process, the consumption of Pb and Cs monomers leads to an insufficient supply of these monomers for the continuous growth of the nuclei, ultimately resulting in the formation of smaller nanoparticles. In contrast, under bromide‐deficient conditions, the scarcity of Br^−^ ions limits the formation of the [PbBr_6_]^4−^ framework and CsPbBr_3_ nuclei. The abundant residual lead or cesium monomers allow these limited nuclei to continue growing, leading to the formation of larger nanoparticles [29]. The research results showed that when the molar ratio of Cs/Pb/Br was 0.1/0.1/3, more cubic CsPbBr_3_ nanocrystals were generated in the composite under the shearing force of an open mill. As shown in Figure 2a, the brominated butyl rubber was initially softened and adhered to the roller surface after being squeezed by the front and rear rollers of an open mill, forming a continuous rubber layer. This layer showed no fluorescence when exposed to a UV lamp with a maximum wavelength of 365 nm and a power of 12 W. After the addition of cesium (CsSt) and lead sources (PbSt_2_), the composite formed after 4 min of mixing emitted a distinct green fluorescence (Figure 2b).

Based on the exploration of vulcanization conditions in the Supporting Information 1.2, it was found that under the sulfidation condition of 100°C for 15 min, more uniform‐sized perovskite quantum dots were synthesized. Through the hot‐pressing process, a flexible quantum dot light conversion film with a large‐area of 17 × 17 cm^2^ and a thickness of 1.19 ± 0.01 mm was successfully fabricated, as depicted in Figure 2c. Under the UV light illumination, the film exhibited uniform green fluorescence, indicating that the CsPbBr_3_ quantum dots generated in situ were uniformly dispersed in the rubber matrix. Figure 2e shows a digital image of the CsPbBr_3_/BIIR composite film. The clear visibility of buildings and trees through the film validates that the high light transmittance of the fabricated CsPbBr_3_/BIIR composite film. The results of further testing on the transmittance of pure BIIR and CsPbBr_3_/BIIR composite are shown in Figure S5a. When the thicknesses of pure rubber and composite materials are 0.35 ± 0.01 mm and 0.33 ± 0.01 mm respectively, the transparency values at 550 nm were 89.8% and 82.1% respectively, indicating that the composite material has high transparency.

The fluorescence distribution of the CsPbBr_3_/BIIR composite film was observed using an inverted fluorescence microscope (ZEISS, Axio Observer 3) with a blue channel laser excitation at 450–490 nm and emission detection at 500–550 nm, as shown in Figure S6. In the control CsPbBr_3_/IIR composite film, there were fluorescence aggregation points caused by the aggregation of quantum dots. However, the CsPbBr_3_/BIIR composite film exhibited uniform green fluorescence under blue light excitation. Additionally, in the bright field, the blocky structures with different sizes were clearly observed in the matrix, which showed no fluorescence under blue light excitation. This was attributed to the presence of unreacted stearate powder. Further, the dispersion of quantum dots in the CsPbBr_3_/BIIR slice was observed using a transmission electron microscopy (TEM). As shown in Figure 2f, the formation of perovskite quantum dots is confirmed by the contrast difference between the quantum dots (dark) and the polymer (light). The quantum dots were randomly dispersed within the polymer matrix. The particle size distribution was determined by analyzing high‐resolution transmission electron microscopy (HR‐TEM) images using Image J software (version 1.53k). More than 100 individual nanoparticles were manually measured to ensure statistical significance. The number‐average diameter was calculated to be 2.56 ± 0.27 nm, and the data were fitted with a log‐normal distribution (Figure S5b). The generated long‐chain IIR‐OOCC_17_H_35_ polymer acted as a robust ligand to suppress surface defects and aggregation of quantum dots, endowing the CsPbBr_3_/BIIR film with excellent optical properties. Additionally, the generated quantum dots are embedded in the amorphous polymer matrix, which can significantly enhance the stability of the perovskite because the polymer molecular chains have good gas barrier properties and resisting the penetration of external water/oxygen. Furthermore, the quantum dots have high crystal quality, with lattice fringes spacing of 0.41 nm (Figure 2g), corresponding to the (110) plane of the cubic CsPbBr_3_ crystal, which is further confirmed by the FFT pattern (Figure 2h) [30]. Elemental mapping analysis of the CsPbBr_3_/BIIR composite film was conducted, and the results are shown in Figure 2i,j. The elements Cs, Pb, Br, and O were uniformly distributed, indicating that the CsPbBr_3_ quantum dots and long‐chain IIR‐OOCC_17_H_35_ polymers were uniformly distributed in the rubber matrix. Additionally, the aggregated nanoparticles correspond to the distribution of Cs, Pb, and Br, indicating that there is a small amount of quantum dot aggregation in the matrix.

**FIGURE 3:**
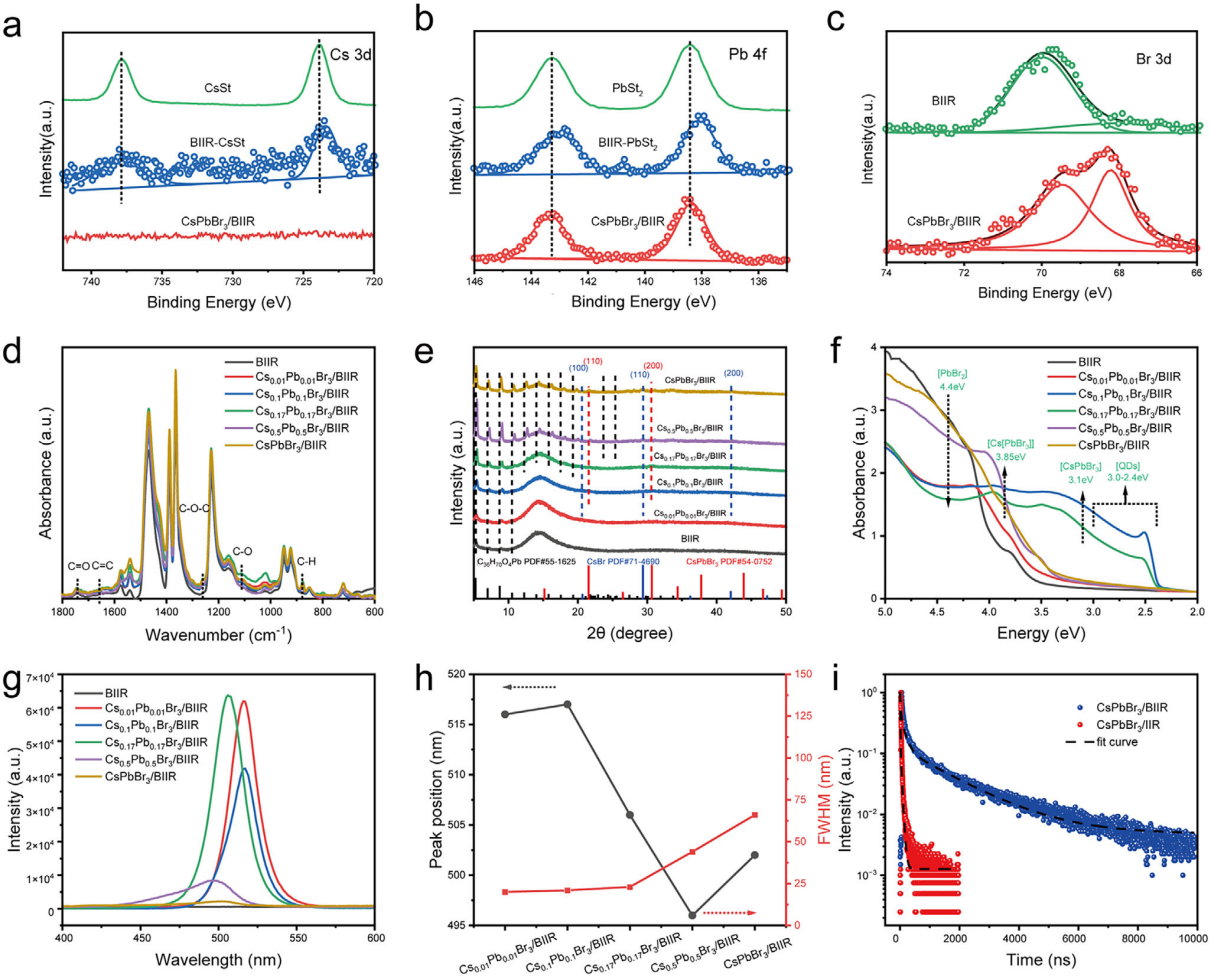
(a) Cs 3d, (b) Pb 4f, and (c) Br 3d high‐resolution XPS spectra of the Cs_0.1_Pb_0.1_Br_3_/BIIR composite film. (d) FTIR spectra of CsPbBr_3_/BIIR composite films with different bromine contents under hot‐pressing at 100°C for 15 min. (e) XRD patterns of different CsPbBr_3_/BIIR composite films hot‐pressed at 100°C for 15 min. (f) Absorption spectra, (g) PL spectra, and (h) the maximum fluorescence peak and FWHM of CsPbBr_3_/BIIR composite films with different bromine contents under hot‐pressing at 100°C for 15 min. (i) PL decay curves and fitting results for CsPbBr_3_/BIIR and CsPbBr_3_/IIR composite films.

The chemical interactions between the brominated butyl rubber and two precursors or perovskite crystals were investigated using an X‐ray photoelectron spectroscopy (XPS)and Fourier transform infrared spectroscopy (FTIR). The elemental valence and composition of the CsPbBr_3_/BIIR composite film with a Cs/Pb/Br molar ratio of 0.1/0.1/3 was further characterized using an XPS spectrometer. Figure S7 shows the electron signals for the Cs 3d, Pb 4f, and Br 3d orbitals, indicating the presence of Cs, Pb, and Br elements in the CsPbBr_3_/BIIR composite. The same result was also confirmed by the elemental analysis of CsPbBr_3_/BIIR composite film with different bromine concentrations (Table S1). Further high‐resolution XPS spectra showed that the binding energies of Cs, Pb, and Br in the CsPbBr_3_/BIIR composite film underwent a significant shift compared to their precursors, indicating that a chemical reaction has occurred in the polymer matrix. After the dissociation of Cs^+^ and Pb^2+^ dissociated from the precursors and the Br^−^ cleavage from the polymer bonds were re‐bonded by ionic bonds to form the PQDs (Figure 3a–c). The characteristic split peaks of Pb 4f are located at 138.4 eV (Pb 4f_5/2_) and 143.4 eV (Pb 4f_7/2_), indicating the presence of Pb^2+^ in the prepared composite (Figure 3b). There are significant changes in the Br 3d high‐resolution spectra of the composite film with the BIIR precursor (predominantly covalent bonds), with two distinct split peaks of Br 3d_3/2_ (69.4 eV) and 3d_5/2_ (68.2 eV) respectively, with an energy difference of 1.2 eV, indicating an oxidation state of −1 for Br in the CsPbBr_3_/BIIR composite [31, 32]. Comprehensive analysis confirmed the successful preparation of cubic phase CsPbBr_3_/BIIR composite films via an open‐mixing shear method.

As shown in Figure S8a, the characteristic peaks at 1576, 1540 cm^−1^ and 1741, 1020 cm^−1^ in the BIIR spectrum corresponded to the carboxylate COO^−^ group and ester additives, which are added as a part of the production ingredients during the production process of commercial BIIR. After mixing with the Cs source or Pb source, the C─O─C stretching vibration peak shifted to 1012 cm^−1^, the C═O stretching vibration peak at 1742 cm^−1^ exhibits a red shift, and the CH_2_ in‐plane rocking vibration peak at 722 cm^−1^ intensified, shifting to 720 and 716 cm^−1^, respectively, indicating a strong interaction between Pb^2+^/Cs^+^ and BIIR. The ═C─H bending vibration peak of the trans‐disubstituted alkene at 850 cm^−1^ became broader and stronger with the addition of Cs or Pb source, indicating a strong interaction between Pb^2+^/Cs^+^ and the BIIR double bonds of BIIR. As shown in Figure 3d, in the BIIR spectrum, the 1655 cm^−1^ peak corresponds to the C═C stretching vibration peak. When either Cs or Pb was added, the peak positions remained unchanged. When both Cs and Pb sources were added simultaneously, the peak position shifted to 1646 cm^−1^, indicating an enhancement of the conjugation effect in the CsPbBr_3_/BIIR composite film, possibly due to the chemical reaction of the allyl bromide structure. The new peaks at 1260 and 1110 cm^−1^ corresponded to the C─O stretching vibration peaks of the ester group, and the C─H out‐of‐plane bending vibration peak of the tri‐substituted alkene at 875 cm^−1^ appeared in the CsPbBr_3_/BIIR composite films, indicating that the long alkyl chain of the stearic acid salt may have been grafted onto the main chain of BIIR through a nucleophilic substitution reaction. This was attributed to the extremely active allyl bromide at the allyl position on the BIIR main chain. In the presence of a catalyst (Cs^+^, Lewis acid Pb^2+^), the C─Br bonds were broken through mechanical shearing or thermal action to form X^−^ ions and carbon positive ion intermediates. Subsequently, driven by their relatively lower formation energy, these ions recombined through ionic bonds to form CsPbX_3_ quantum dots, while the carboxylate ion (COO^−^) attacked the carbon positive ion intermediate, forming allyl ester (IIR‐OOCC_17_H_35_). When the Cs:Pb:Br molar ratio was 1:1:3, new peaks appeared at 1314 and 1297 cm^−1^ in the CsPbBr_3_/BIIR composite film, most likely caused by the red shift of the symmetric stretching vibration of the COO^−^ group due to the cation‐carboxylate interaction in the excess stearate.

Figure 3e shows the XRD patterns of CsPbBr_3_/BIIR composite films with different Br contents after hot‐pressing at 100°C for 15 min. The broad peak in the XRD curves between 10° and 20° corresponded to the amorphous diffraction peak of BIIR. As the amount of Cs and Pb sources increased, the amorphous diffraction peak of BIIR gradually decreased, the diffraction peaks of the Pb source became stronger, and their positions red‐shifted to 5.3°, 7.1°, 9.0°, 10.8°, 12.6°, 14.3°, 16.2°, 18.0°, 19.8°, 23.5°, and 25.4°, respectively. To further analyze the reason, BIIR was mixed uniformly with either the cesium source or the lead source separately, hot‐pressed at 100°C for 15 min, and their XRD patterns were tested. As shown in Figure S8b, crystalline peaks appeared at 5.4° and 9.1°in the BIIR curve. These crystalline peaks were likely due to the interaction between stearate ions and bromine atoms on the BIIR molecular chains. This is because commercial BIIR uses calcium/zinc stearate as a part of their production formulation. After adding the cesium source, the crystalline peaks in the BIIR‐CsSt curve appeared at 5.47°, 9.1°, and 9.526°. After adding the lead source, the crystalline peaks in the BIIR‐PbSt_2_ curve appear at 5.51°, 9.2°, and 9.53°, indicating that stearic acid lead has a stronger interaction with the molecular chains of BIIR than with stearic acid cesium. As the amounts of cesium and lead precursors increased, the diffraction peaks of the lead source showed varying degrees of red shift. This might be due to the formation of [PbBr_6_]^4−^ octahedra by the lead source and the allylic bromine, or the formation of C═O…Pb strong coordination interaction between the CsPbBr_3_ QDs and the generated allyl ester. When the molar ratio of Cs:Pb:Br increased to 0.5:0.5:3, due to insufficient bromide ion concentration in the system, diffraction peaks corresponding to the reaction intermediate CsBr (PDF#71‐4690) appear at 20.6° (100), 29.5° (110), and 42° (200). This was likely generated by a nucleophilic substitution reaction between the allylic bromine on the BIIR molecular chains and the cesium source. With further increases in lead and cesium precursors, diffraction peaks for cubic CsPbBr_3_ (PDF#54‐0752) appear at 21.6° and 30.6°, corresponding to the (110) and (200) planes, respectively [33].

Further observing the luminescence properties of CsPbBr_3_/BIIR composite films with different bromine contents at a vulcanization temperature of 100°C, their absorption and photoluminescence (PL) spectra are shown in Figure 3f,g, respectively. In a bromine‐rich environment (Br/Cs molar ratio >18), the first excitonic peak of the composite films showed significant enhancement, and the PL peak of the composite films exhibited a red shift of 4 nm compared to the non‐vulcanized CsPbBr_3_/BIIR composite films. Optical images under sunlight and UV light irradiation show a similar trend, with the film color turning greener and increasing fluorescence intensity. This is mainly due to the thermodynamic reactions under a 15T platen pressure during flat‐plate vulcanization, which promoted further lateral growth of perovskite quantum dots, resulting in larger particles [34, 35]. Notably, when the Br/Cs molar ratio is 18, a distinct sharp peak is observed at 3.95 eV, and the first exciton peak of the composite films shifts to 2.52 eV. This indicated that the composite film underwent further thermodynamic reactions at a vulcanization temperature of 100°C, generating cubic‐phase CsPbBr_3_ and Cs_4_PbBr_6_ nanocrystals. However, in composite films with low bromine content treated under vulcanization conditions, the PL peak of the composite films shifted from 505 nm to 496 nm (Figure 3h). This was because the growth of the quantum dot nuclei formed by mechanical shearing could not obtain sufficient bromine source supply, and a large amount of residual stearic acid lead dissolved to form a high concentration of Pb^2+^, which might cause the nucleophilic substitution reaction to proceed in the reverse direction. Some perovskite quantum dots decomposed into CsPbBr_3_ nuclei, and might have further decomposed to generate intermediates CsBr and PbBr_2_.

Time‐resolved photoluminescence spectroscopy was used to characterize the exciton recombination dynamics of CsPbBr_3_/BIIR and CsPbBr_3_/IIR composite films. Figure 3i shows the PL decay curves of the CsPbBr_3_/BIIR and CsPbBr_3_/IIR composite films fitted with a tri‐exponential decay Equation S1. The average fluorescence lifetime was calculated using the Equation S2. Related studies showed that short fluorescence lifetime component (*τ*_1_) originates from non‐radiative recombination mediated by defect states, such as bromine vacancy (V_Br_) or lead interstitial (Pb_i_), while *τ*_2_ and *τ*_3_ correspond to radiative recombination of localized or self‐trapped excitons and free band‐edge excitons, respectively [36]. From Table S2, compared to the CsPbBr_3_/IIR composite film, CsPbBr_3_/BIIR shows an ultra‐long decay lifetime of 1189.73 ns, mainly originating from its lower surface defect density. This is attributed to the brominated butyl rubber molecular chains providing an almost perfect physical confinement and chemical passivation environment for the quantum dots, while effectively isolating external degradation factors. Therefore, the CsPbBr_3_/BIIR composite film exhibited a high PL quantum yield (91.89% under UV light and 41.3% under blue light irradiation, Figure S9) and a decay lifetime of 1189.73 ns. This indicates that using the polymer chains as the bromine source not only promotes the effective encapsulation of the polymer for the perovskite nanocrystals, but also effectively inhibits the formation of defects, thereby preparing high‐performance CsPbBr_3_/BIIR composite films.

### 2.3 Kinetic Reaction Mechanism for CsPbBr_3_/BIIR Composite Films

To understand the potential mechanism and driving force of the mechanochemical synthesis of CsPbBr_3_/BIIR composites, it was necessary to systematically track the properties of the synthesized perovskite compounds over time. The following conditions should be met: (i) Uniform force should be applied to the precursors throughout the reaction process, maintaining a fixed roll speed ratio of 1:1.35; (ii) The reaction temperature should be kept constant, with the roller surface temperature maintained at 25°C via circulating cooling water to achieve a quasi‐dynamic process. To further identify the main contributors to the nucleation process, we designed experiments in which the halogenated butyl rubber, cesium source, and lead source served as the sole sources of halogen, lead, and cesium, respectively. By separating the three sources, this helps us understand and establish the quantum dot formation mechanism. While maintaining the same amounts of halogenated butyl rubber, lead salt, and cesium salt, the order of addition of the lead salt and cesium salt was adjusted to observe quantum dot growth under different shearing times.

**FIGURE 4:**
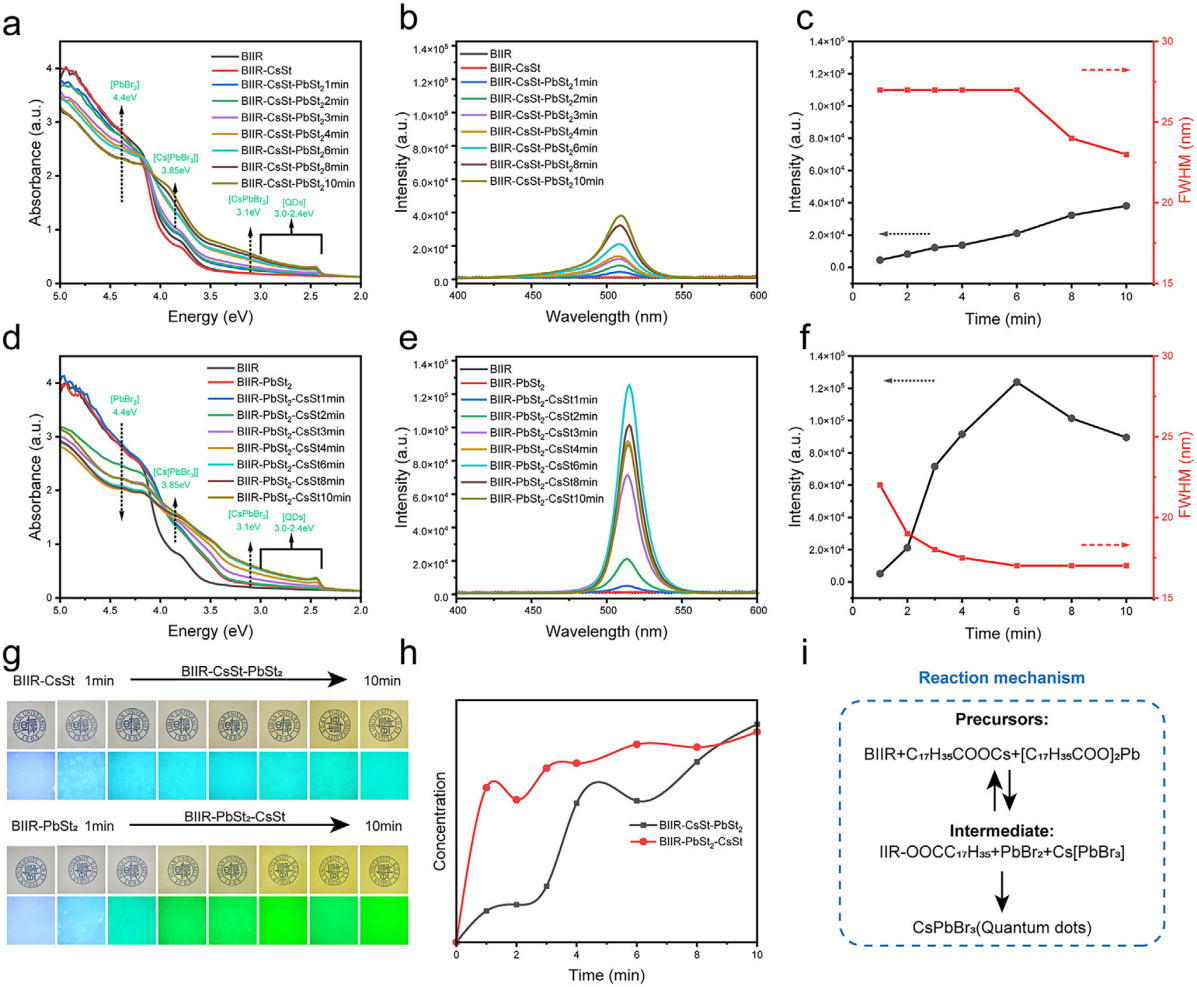
(a) Absorption spectra, (b) PL spectra, and (c) the maximum fluorescence intensity and FWHM of CsPbBr_3_/BIIR composite films with the first addition of cesium source. (d) Absorption spectra, (e) PL spectra, and (f) the maximum fluorescence intensity and FWHM of CsPbBr_3_/BIIR composite films with the first addition of lead source. (g) Optical images and fluorescence photographs under UV lamp (365 nm, 40 W) of CsPbBr_3_/BIIR composite films with different Cs/Pb addition sequence at different time intervals. Cs[PbBr_3_] intermediate growth curves (h) and reaction pathways (i) of CsPbBr_3_/BIIR composite films under different Cs/Pb addition sequences.

Figure 4 shows the absorption spectra, photoluminescence (PL) spectra, and changes in the PL intensity at the maximum emission peak and a narrow full width at half maximum (FWHM) for the samples taken at different open‐mixing time. As shown in Figure 4a, the characteristic peak at 3.79 eV primarily corresponds to the π→π* electronic transition of the conjugated diene structure (─C═C─C═C─) in the BIIR molecular chains. First, a cesium source was added, and the absorption value at 3.79 eV decreased slightly. This might be because the cesium ions (Cs^+^) in the Cs source possessed strong electropositivity and electron‐donating ability, causing the electron cloud of the conjugated diene structure to shift toward the Cs^+^, which made the C─Br bond extremely vulnerable. Subsequently, Pb salt was added to the above system, and a sharp excitonic peak appeared at 2.45 eV in the absorption spectra of the composite films, corresponding to a PL peak at 508 nm, indicating the rapid formation of CsPbBr_3_ nanocrystals via an open‐mixing shear (Figure 4b). As the open‐mixing time increased, the intensities of the excitonic peak at 2.45 eV and the PL peaks increased linearly, indicating the instantaneous generation of more CsPbBr_3_ nanocrystals under continuous shear driving (Figure 4c). When the open‐mixing time reached 8 min, the absorption peak at 3.79 eV nearly disappeared, and a sharp peak appears at 3.91 eV, accompanied by a decrease in the FWHM of the film. This suggests the possible formation of Cs_4_PbBr_6_ nanocrystals and smaller cubic‐phase CsPbBr_3_ nanocrystals, attributed to the ^1^S_0_→^3^P_1_ transition of the Pb^2+^ center, which is consistent with the optical properties of Cs_4_PbBr_6_ reported in the literature [29, 37].

When the addition sequence was changed, and the Pb source was first added to the BIIR, the maximum absorption peak of BIIR shifted from 3.54 to 3.3 eV (Figure 4d). According to the theory of Hard‐Soft Acid‐Base theory, the Pb^2+^ in the stearic acid lead (a soft acid) had a strong affinity for Br^−^, easily forming stable coordination bonds, which may lead to the formation of a complex (e.g., [PbBr_4_]^2−^ tetrahedra, [PbBr_6_]^4−^ octahedra) with lead as the center and bromine as the ligand. Upon further addition of the Cs salt, cesium ions diffused into the voids, naturally forming uniform perovskite quantum dots. After 2 min of kneading, a sharp excitonic peak appears at 2.44 eV in the absorption spectrum of the composite film, corresponding to a PL peak at 515 nm, indicating that the pre‐formed Pb‐Br complex can promote the rapid generation of cubic‐phase CsPbBr_3_ nanocrystals under shear force‐driven conditions (Figure 4e). With the extension of the kneading time, the PL intensity of the composite film at 2.44 eV increases exponentially. After kneading for 6 min, the PL intensity of the composite film reached its maximum, and the corresponding excitonic peak also reached the highest value. It is worth noting that the CsPbBr_3_ composite film (FWHM 17 nm) synthesized in situ by the pre‐formed Pb‐Br complex has a narrower half‐width than that (FWHM 23 nm) synthesized by adding a Cs source first, approaching high‐quality quantum dots synthesized by a thermal injection method. [38, 39, 40] This indicates that the mechanical chemical synthesis of high‐quality CsPbBr_3_ nanocrystals can be achieved by simply changing the addition order of the precursors (Figure 4f). Therefore, the pre‐formation of the lead‐bromine complex is crucial for improving the uniformity of quantum dot nucleation. The growth rate of quantum dots can be further controlled by the diffusion of Cs ions. This phenomenon is more intuitively observed through optical photographs and fluorescence photographs under UV lamp (365 nm, 40 W) irradiation (Figure 4g). For the composite film with initially added the Cs salt, it was observed that as the shearing time increased, the film maintained a high degree of transparency, its color gradually turned yellow, and the corresponding fluorescence intensity gradually increased. In contrast, the composite film with the first added Pb salt showed obvious green fluorescence after kneading for 2 min, and the green fluorescence intensity quickly reached the maximum value.

To investigate the growth curve of the quantum dots and their reaction mechanism, we conducted a further analysis of the above absorption spectra (Figure 4h,i). When the brominated butyl rubber (BIIR) was first mixed with the Cs source, the changes are minimal (Figure 4a). After adding the Pb source, the broad absorption peak around 4.2–5 eV, possibly corresponding to the stearate salt[BIIR] complex, gradually decreased with prolonged shear time, which may be due to the consumption of stearate salt. The intensity change allowed us to monitor the depletion of stearate salt during the reaction process. Meanwhile, an additional peak appears at lower energy (3.85 eV), which was attributed to the formation of the PbBr_3_^−^ complex. Once the accumulation of PbBr_3_^−^ exceeds the nucleation threshold (supersaturation), the CsPbBr_3_ quantum dots are formed. For the case where the Pb source was added first, we observed a significant enhancement in the intensity of the 3.5–4.0 eV region, likely due to the formation of the Pb‐Br complex between BIIR and the Pb source (e.g., [PbBr_4_]^2−^ tetrahedra, [PbBr_6_]^4−^ octahedra). Upon further addition of the Cs salt, the intensity of 4.5–5 eV region gradually decreased with prolonged shearing time, while the intensities of 3.5–4.0 eV and 2.4–3.0 eV regions were significantly increased, and a new peak appeared at 3.79 eV, which was caused by the formation of PbBr_3_^−^ complex and quantum dot generation.

Because the time for the PbBr_3_^−^ complex to bind with Cs^+^ ions is on the order of seconds, we assumed this period to be 0, and the growth of quantum dots was reflected by monitoring the concentration of the Cs[PbBr_3_] intermediate, as shown in Figure 4h. The QD concentration in the BIIR‐PbSt_2_‐CsSt composite film increased rapidly at the beginning of the reaction and then grew slowly in a linear manner. The QD concentration in the BIIR‐CsSt‐PbSt_2_ composite film basically increased linearly with increasing the reaction time. From the comparison of different addition sequences, it is evident that the pre‐formation of the PbBr_3_^−^ complex is a prerequisite for a narrow size distribution. When cesium ions diffuse into the void, the complex enables the rapid and uniform nucleation and growth of the perovskite quantum dots. For the system in which stearic acid cesium is pre‐mixed in the brominated butyl rubber, the added stearic acid lead couples with bromide ions under mechanical shear force to form the PbBr_3_^−^ complex, and then quickly combines with the cesium ions in the matrix to form the CsPbBr_3_ nanocrystals. Therefore, the sequence of Cs/Pb addition directly affected the uniformity of nucleation and the difference in growth speed. Additionally, during the mechanical shear reaction process, we observed a new peak at 4.4 eV, indicating that a brominated lead intermediate was generated in a bromine‐rich environment [41]. Further speculation of the mechanism from the precursor to the formation of quantum dots is shown in Figure 4i.

### 2.4 Tunable Optical and Mechanical Properties of CsPbX_3_/HIIR Composite Films

**FIGURE 5:**
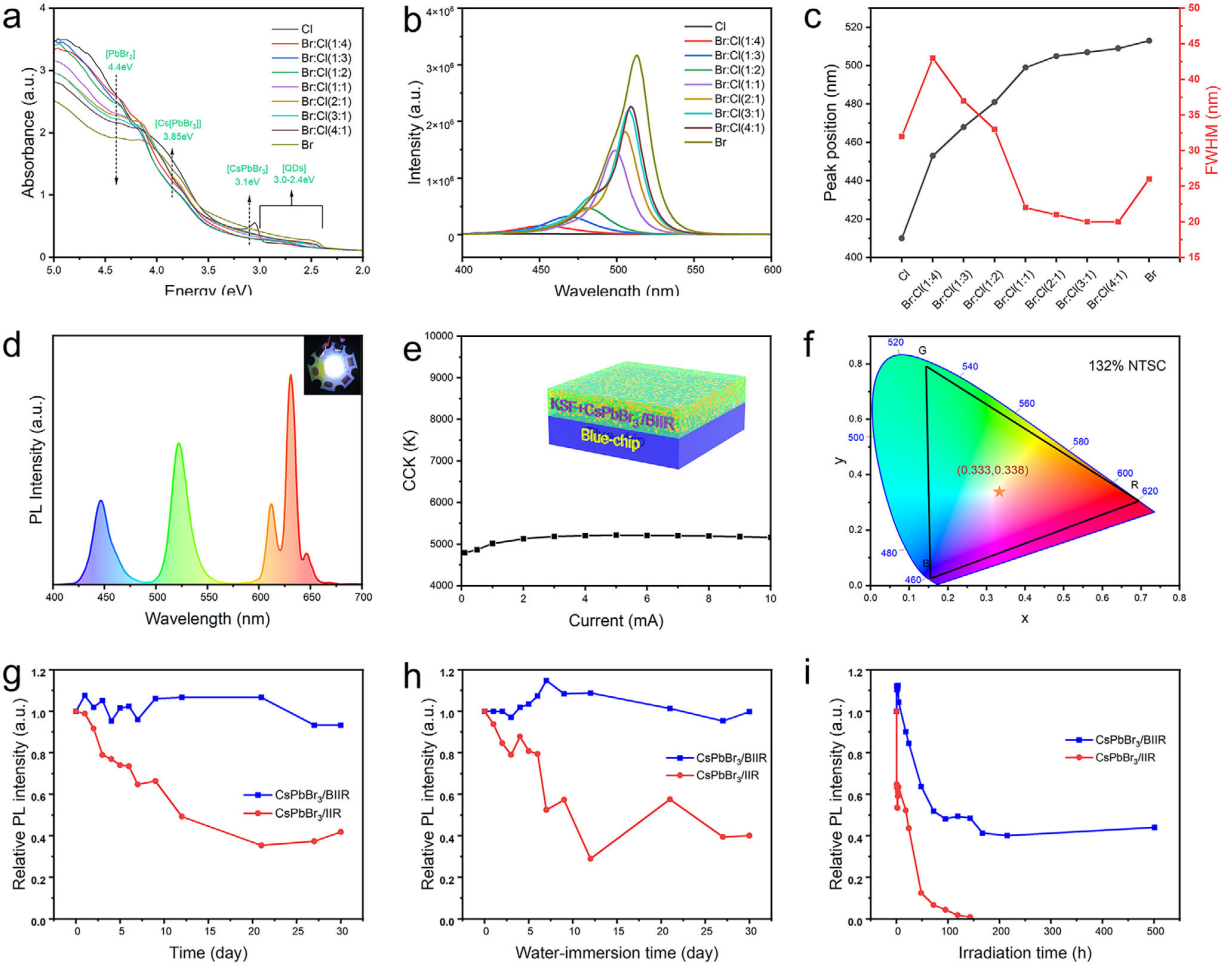
(a) Absorption spectra, (b) PL spectra, and (c) the maximum fluorescence peak and FWHM of the CsPbX_3_/HIIR composite films with different halogen ratios. (d) PL spectrum of the WLED device at a drive current of 1 mA. (e) CCT variation of the WLED device under different driving currents and schematic diagram of the KSF+CsPbBr_3_/BIIR film for WLED device. (f) color gamut range of WLED device constructed with CsPbBr_3_/BIIR composite films. (g) Storage stability, (h) water resistance stability, and (i) blue light irradiation stability for CsPbBr_3_/BIIR and CsPbBr_3_/IIR composite films.

The valence band maximum of all‐inorganic CsPbX_3_ perovskites is composed of an anti‐bonding orbital formed by the hybridization of the 6s orbital of Pb^2+^ and the np orbital of X^−^ (where n = 3 for Cl^−^, n = 4 for Br^−^), where the p orbital of the halide ion playing a dominant role. Therefore, changing the halide composition is an effective way to tune the optical properties of the perovskite nanocrystals. In this study, brominated butyl rubber and chlorinated butyl rubber were used as the bromine and chlorine sources, respectively. The emission wavelength of the perovskite composite film can be effectively controlled by systematically adjusting the Br/Cl ratio. Rubber with different halogen ratios was mixed, and the Cs and Pb precursors were added at a molar ratio of Cs/Pb/Br (0.1/0.1/3) for the in situ reaction. The absorption spectra and fluorescence spectra of the composite films with different halogen ratios are shown in Figure 5a–c.

As shown in Figure 5a,b, the absorption peak of the composite film with pure chlorinated butyl rubber was located at 406 nm, caused by the excitonic emission of CsPbCl_3_ quantum dots. The emission peak was observed at 410 nm, with a FWHM of 32 nm and a Stokes shift of 4 nm. As the bromine molar ratio increased, the absorption peak was continuously red‐shifted, and the emission wavelength of the corresponding composite film was tunable from 410 to 513 nm (Figure 5c). For the composite film with pure brominated butyl rubber, the absorption peak red‐shifted to 496 nm, caused by the excitonic emission of CsPbBr_3_ quantum dots. The emission peak was at 513 nm, with a FWHM of 26 nm and a Stokes shift of 17 nm. The band gap was further calculated using the Planck‐Einstein formula. The calculated band gap for the CsPbCl_3_ composite film was 3.05 eV. As the Br content increases, the band gap of the composite film continuously decreased. This is because the band gap of the composite film is influenced by the halogen ion radius. The larger the halogen ion radius (*R_Cl_* < *R_Br_*), the smaller the band gap.

As shown in Figure S10a–c, the CsPbBr_3_/BIIR composite film was repeatedly bent at various angles without exhibiting cracking or fracture, indicating its excellent flexibility and potential for application in multifunctional flexible optoelectronic devices. Further mechanical performance tests of CsPbBr_3_/BIIR composite films with different bromine contents (Figure S11d) revealed that all films remained uncrosslinked with consistent mechanical properties, demonstrating that the different amounts of stearic acid precursors had no impact on the mechanical performance of BIIR, and the generated CsPbBr_3_ quantum dots had minimal influence on the mechanical characteristics of the matrix. BIIR, composed of an isobutylene‐isoprene copolymer, features a highly saturated backbone in which the isoprene segments are flanked by symmetrical methyl groups, resulting in a very small free volume. This structure endows it with significantly better gas barrier properties than other general rubber. Figure S11e,f displays the hydrophilicities of the pure BIIR and CsPbBr_3_/BIIR composite film surfaces, respectively. Pure BIIR exhibited hydrophobicity, with a contact angle of 103.77°. After undergoing an ionic reaction, the CsPbBr_3_/BIIR composite film remained hydrophobic, with its θ value changing to 101.72°, which can effectively prevent the erosion of external water and oxygen on the internal CsPbBr_3_ QDs.

### 2.5 Application and Stability Study of CsPbBr_3_/BIIR Composite Films

Based on the highly efficient and stable green CsPbBr_3_/BIIR composite film, combined with commercial KSF (K_2_SiF_6_:Mn^4+^) red phosphor, a white LED device was constructed by coating it onto a blue chip (λ_d_ = 450 nm). Figure 5d shows the photoluminescence (PL) spectra of the device, evidencing three distinguishable emission band sets from a 450 nm blue chip, a 522 nm CsPbBr_3_/BIIR composite, and a 631 nm KSF phosphor. The fabricated WLED exhibited bright white emission at a driving current of 1 mA in the inset Figure 5d. The inset in Figure 5e displays the structural schematic of the WLED device, which consists of KFT+CsPbBr_3_/BIIR film (green and red conversion layer) and commercial InGaN blue LED chip. As the drive current increased, the EL intensity of the device gradually increased, while its CCT showed no significant changes (Figure 5e), indicating that the packaged white LED device had good luminescence performance and stability. The Commission International L'Eclairage (1931CIE) chromaticity coordinates of the WLED were (0.333, 0.338), which were close to the standard white light coordinates (0.33, 0.33). Simultaneously, this white LED device achieved a correlated color temperature (CCT) of 5200 K, and a color gamut range up to 132% of the NTSC standard color gamut (Figure 5f). Compared with the previously reported perovskite‐organic polymer composite (Table S3), our prepared CsPbBr_3_/BIIR composite film displays a relatively high color purity, wide color gamut, and excellent stability against air and water exposure, which makes them promising for solid‐state lighting and liquid crystal displays.

To systematically investigate the stability of CsPbBr_3_/BIIR composite films under different conditions, such as room temperature, water, blue light (450 nm, 5000 nit), and heat, high‐performance perovskite quantum dots synthesized by a hot‐injection method were mixed with butyl rubber to obtain CsPbBr_3_/IIR composite films as a control group. From Figure 5g, after storage in an open environment for 30 days, the PL intensity of the control group decreased to 42% of its initial value, whereas the luminescence intensity of the CsPbBr_3_/BIIR composite film showed no significant fluctuation, still maintaining > 90%. This indicates that the strong interaction between the QDs and the rubber molecular chains significantly improve the storage stability of the CsPbBr_3_/BIIR composite film. From Figure 5h, compared to the CsPbBr_3_/IIR composite film, after continuous immersion in water for up to 30 days, the PL intensity of the CsPbBr_3_/BIIR composite film remained as high as 99.8% of its initial value, indicating the extremely excellent long‐term water resistance stability of the CsPbBr_3_/BIIR composite film. Furthermore, after continuous blue light irradiation for 500 h, the luminescence intensity of the CsPbBr_3_/BIIR composite film still maintains 44% of its initial value, while its emission peak position showed no significant change, indicating good blue light irradiation resistance of the CsPbBr_3_/BIIR composite film (Figure 5i). On the one hand, CsPbBr_3_ quantum dots were in situ synthesized within the BIIR matrix, enabling the polymer molecular chains to rapidly and effectively encapsulate the generated QDs, thereby preventing the erosion of water and oxygen from the external environment on the internal QDs. On the other hand, the acrylate structure generated by the reaction between BIIR and stearate forms an interfacial passivation layer on the quantum dot surface. This structure has a strong interaction with the C═O…Pb coordination in the quantum dots, successfully reducing the surface defects of the perovskite film and inhibiting non‐radiative recombination loss. After thermal cycling tests between 30°C and 100°C, the sample still maintained 53% of its initial value (Figure S11a). After a further 5 cycles, the fluorescence performance stabilized at 46% of the initial value (Figure S11b), indicating good thermal stability of the CsPbBr_3_/BIIR composite film.

## 3 Conclusions

In conclusion, we established a novel one‐step, solvent‐free mechanochemical strategy for the in situ synthesis of CsPbX_3_ (Br/Cl) quantum dots within a halogenated butyl rubber (HIIR) matrix via open‐mill shear. This approach enables the uniform embedding of monodisperse 2.6 nm CsPbBr_3_ QDs in a topologically entangled network, where the confined environment—under the synergistic catalysis of Cs^+^/Pb^2+^ and mechanical shear—drives high‐density homogeneous nucleation through an ionic reaction pathway. The resulting CsPbX_3_/BIIR composite exhibited exceptional optical properties, including a narrow emission at 515 nm (FWHM = 17 nm), a PLQY of 91%, and an ultralong lifetime of 1189 ns. Importantly, the in situ formation of IIR‐OOCC_17_H_35_ chains creates an interfacial allyl ester passivation layer that effectively suppresses surface traps and blocks the ingress of H_2_O/O_2_, endowing the composite film with outstanding environmental stability: it maintained over 90% of its initial intensity after 30 days in ambient air, 99.8% after 30‐day water immersion, and 44% after 500 h of blue‐light irradiation. When integrated into flexible white LEDs, the composite delivered standard white emission with CIE coordinates of (0.333, 0.338), a color rendering index of 55.7, and a wide color gamut covering 132% of the NTSC standard. Mechanistic studies revealed that the pre‐formation of the PbBr_3_^−^ complex under bromide‐rich conditions controlled by precursor sequence and halogen concentration is essential for achieving narrow‐size‐distributed, high‐performance PQDs, providing key insights into growth kinetics. This work not only offers a low‐cost and high‐efficiency preparation route for high‐quality perovskite nanocrystals but also demonstrates their great potential in flexible optoelectronics, including displays, X‐ray scintillators, and wearable luminescent textiles.

## 4 Experimental Section

### 4.1 Materials

All the chemicals were of analytical grade. Commercial brominated butyl rubber 2030 (bromine content: 1.8 wt%) and chlorinated butyl rubber 1240 (chlorine content: 1.25 wt%) were purchased from Lanxess, Germany. Cesium stearate (CsSt, 99%) and lead stearate (PbSt_2_, 98%) were purchased from D&B Chemicals Co., Ltd. Butyl rubber 268S was obtained from ExxonMobil, USA. An open mill (model: CREE‐6015B‐6) and a fully automatic flat‐plate vulcanizing machine (model: CREE‐6014FSC) were manufactured by Dongguan CREE Instruments Technology Co., Ltd.

### 4.2 Synthesis of CsPbBr_3_/BIIR Composite

At room temperature, 100 g of butyl rubber was mixed with different proportions of cesium stearate and lead stearate, and an ionic reaction occurred under the shearing force of an open mill. The specific conditions were as follows: the speed of the front roller was 10 rpm, the speed of the rear roller was 13.5 rpm, the distance between the baffles was 15 cm, and the roller spacing was 1 mm. The molar ratio of Cs:Pb:Br was maintained at x:x:3, with x = 0.01, 0.1, 0.17, 0.5, and 1. Then, the optimal vulcanization temperature was determined by vulcanizing at different temperatures (30°C, 50°C, 80°C, 90°C, 100°C, 110°C, and 120°C) using an automatic flat‐plate vulcanizing machine, with a pressure of 15 T. The obtained perovskite/BIIR composite films were pressed into sheets of dimensions of 60 mm (length) × 60 mm (width) × 1.2 mm (thickness). All processes were carried out in an air atmosphere. The specific components and amounts of the composite films are shown in Table 1. A control sample was prepared by mixing 100 g of butyl rubber with 1 mL of CsPbBr_3_ quantum dot solution (100 mg/mL) synthesized using a hot injection method for 10 min in an open mill to obtain a butyl rubber/perovskite composite film.

**TABLE 1:** Components and contents of CsPbBr_3_/BIIR composite.

Film	Component	BIIR (g)	CsSt (mmol)	PbSt_2_ (mmol)
0#	BIIR	100	0	0
1#	Cs_0.01_Pb_0.01_Br_3_/BIIR	100	0.075	0.075
2#	Cs_0.1_Pb_0.1_Br_3_/BIIR	100	0.75	0.75
3#	Cs_0.17_Pb_0.17_Br_3_/BIIR	100	1.25	1.25
4#	Cs_0.5_Pb_0.5_Br_3_/BIIR	100	3.75	3.75
5#	CsPbBr_3_/BIIR	100	7.5	7.5

The detailed preparation process of the control sample is as follows. First, the CsPbBr_3_ quantum dot solution was synthesized via a reported hot‐injection method [42]. 100 g of butyl rubber and 1 mL of CsPbBr_3_ quantum dot solution (100 mg/mL) were then mixed for 10 min in an open mill to obtain a butyl rubber/perovskite composite film.

To further investigate the influence of different precursors on the mechanochemical generation of perovskite quantum dots, the addition sequence of cesium stearate and lead stearate was changed. The open mill conditions were maintained as follows: the speed of the front roller was 10 rpm, the speed of the rear roller was 13.5 rpm, the distance between the baffles was 15 cm, and the roller spacing was 1 mm. First, 100 g of butyl rubber was mixed with cesium stearate (0.75 mmol) for 10 min, and then lead stearate (0.75 mmol) was added for shearing and mixing to obtain a BIIR‐CsSt‐PbSt_2_ composite. Samples were taken at different times (1, 2, 3, 4, 6, 8, and 10 min) to observe their absorption and fluorescence spectra. Additionally, 100 g of butyl rubber was mixed with lead stearate (0.75 mmol) for 10 min, and then cesium stearate (0.75 mmol) was added for shearing and mixing to obtain a BIIR‐PbSt_2_‐CsSt composite as a control group.

### 4.3 Synthesis of CsPbBr_x_Cl_3‐x_/HIIR Composite

Taking the CsPbBrCl_2_ rubber composite as an example, the total amount of rubber was controlled at 200 g. 87.74 g of bromobutyl rubber (BIIR) and 112.26 g of chlorobutyl rubber (CIIR) were taken, and the corresponding cesium stearate (1.98 mmol) and lead stearate (1.98 mmol) were added at a Cs/Pb/Br molar ratio of 0.1/0.1/3. The shearing reaction was performed on an open mill for 10 min under the following conditions: the speed of the front roller was 10 rpm, the speed of the rear roller was 13.5 rpm, the distance between the baffles was 20 cm, and the roller spacing was 1.5 mm. Subsequently, the material was vulcanized at 100°C for 15 min under a pressure of 15 T using an automatic flat‐plate vulcanizing machine to obtain a CsPbBrCl_2_ rubber composite film, with dimensions of 60 mm (length) × 60 mm (width) × 1.2 mm (thickness). Moreover, by changing the amounts of bromobutyl rubber and chlorobutyl rubber, the CsPbCl_x_Br_3‐x_ composite with different halogen compositions could be obtained using the above preparation process. The specific components and amounts of the composite films are listed in Table 2.

**TABLE 2:** Components and contents of CsPbBr_x_Cl_3‐x_ composite.

Film	Component	BIIR (g)	CIIR (g)	CsSt (mmol)	PbSt_2_ (mmol)
Cl	CsPbCl_3_		200	2.35	2.35
1:4	CsPbBr_2.4_Cl_0.6_	56.19	143.81	2.11	2.11
1:3	CsPbBr_2.25_Cl_0.75_	68.51	131.49	2.06	2.06
1:2	CsPbBrCl_2_	87.74	112.26	1.98	1.98
1:1	CsPbCl_1.5_Br_1.5_	121.97	78.03	1.83	1.83
2:1	CsPbBr_2_Cl	151.53	48.47	1.71	1.71
3:1	CsPbBr_0.75_Cl_2.25_	164.84	35.16	1.65	1.65
4:1	CsPbBr_0.6_Cl_2.4_	172.42	27.58	1.62	1.62
Br	CsPbBr_3_	200		1.5	1.5

### 4.4 Material Characterization and Testing

The morphology of quantum dots, their distribution and elemental mapping in the composite films were observed using an a field‐emission transmission electron microscope (JEOL, JEM F200). The samples were then sliced into ultrathin slices with approximately 50 nm thickness. The crystal structures of the samples were examined using an X‐ray diffractometer (XRD, Bruker D8 Advance) at a scanning speed of 10° min^−1^ in the 5–90° range. The group changes in the samples were analyzed using a Fourier transform infrared spectrometer (FTIR, Thermo, Nicolet iS20) at the range of 500–4000 cm^−1^. The elemental composition and valence states of specific elements in the composite films were determined by an X‐ray photoelectron spectroscopy (XPS, Thermo, Escalab 250Xi), with a sampling depth of 1–10 nm, calibrated using the C 1s peak at 284.8 eV. The absorption spectra of the samples were measured using a UV‐visible spectrophotometer (Shimadzu, UV 2600i) in the scanning range of 200–800 nm. The fluorescence properties of the samples were characterized using a fluorescence spectrophotometer (Shimadzu, RF‐6000) in the detection range of 300–700 nm. The variable‐temperature fluorescence spectra of the samples were obtained using a fluorescence spectrometer (Edinburgh Instruments, FLS 1000) equipped with a liquid nitrogen cryostat (Oxford DN2). The relative spectral power distribution, chromaticity coordinates, correlated color temperature, color rendering index, and optical radiation power of the WLED devices were measured using a photoelectric comprehensive measurement system (PCE series, Hangzhou Everfine Photo‐E‐Info Co., Ltd). The mechanical properties of the composite materials were evaluated using an electronic tensile testing machine (Instron 4465) at a tensile speed of 500 mm/min.

### 4.5 Preparation of White Light‐Emitting Diode Devices

To fabricate of the WLED device, 100 g of BIIR was reacted with cesium stearate (0.75 mmol) and lead stearate (0.75 mmol) in an open mill for 4 min. Then, 13.5 g of K_2_SiF_6_:Mn^4+^ was added, and the mixture was further kneaded for 6 min to ensure thorough mixing. Subsequently, the mixture was compression‐molded into a uniform composite film using a plate vulcanizing machine at 120°C under a 15 T pressure for 5 min. Finally, the CsPbBr_3_/BIIR film was adhered to the surface of a blue light chip (λ_d_ = 450 nm) to encapsulate the WLED device.

## Conflicts of Interest

The authors declare no conflicts of interest.

## Supporting information




**Supporting File**: advs73830‐sup‐0001‐SuppMat.docx.

## Data Availability

Research data are not shared.
