# Supplementary material for: Large‐Scale In Situ Formation Perovskite Quantum Dots/Elastomer Composite for High‐Performance White Light‐Emitting Diodes
Source: Adv Sci (Weinh). 2026 Jan 27;13(17):e22934. doi: 10.1002/advs.202522934 (PMC13042985; doi:10.1002/advs.202522934)
Supplement: Supplementary file 1 — Supporting File: advs73830‐sup‐0001‐SuppMat.docx. [file ADVS-13-e22934-s001.docx]

Supporting Information

Large-Scale In-Situ Formation Perovskite Quantum Dots/Halogenated Butyl Rubber Composite with High Performance for WLED Applications

Yuxian Su, Shirong Yu, Hao Shen, Dongdong Kang, Beibei Wang, Xuebin Yu^*^, and Yongyin Kang^*^

**1.1** **Kinetic Reaction Mechanism of CsPbBr_3_/BIIR Composite Films with** **Different Bromine Contents**


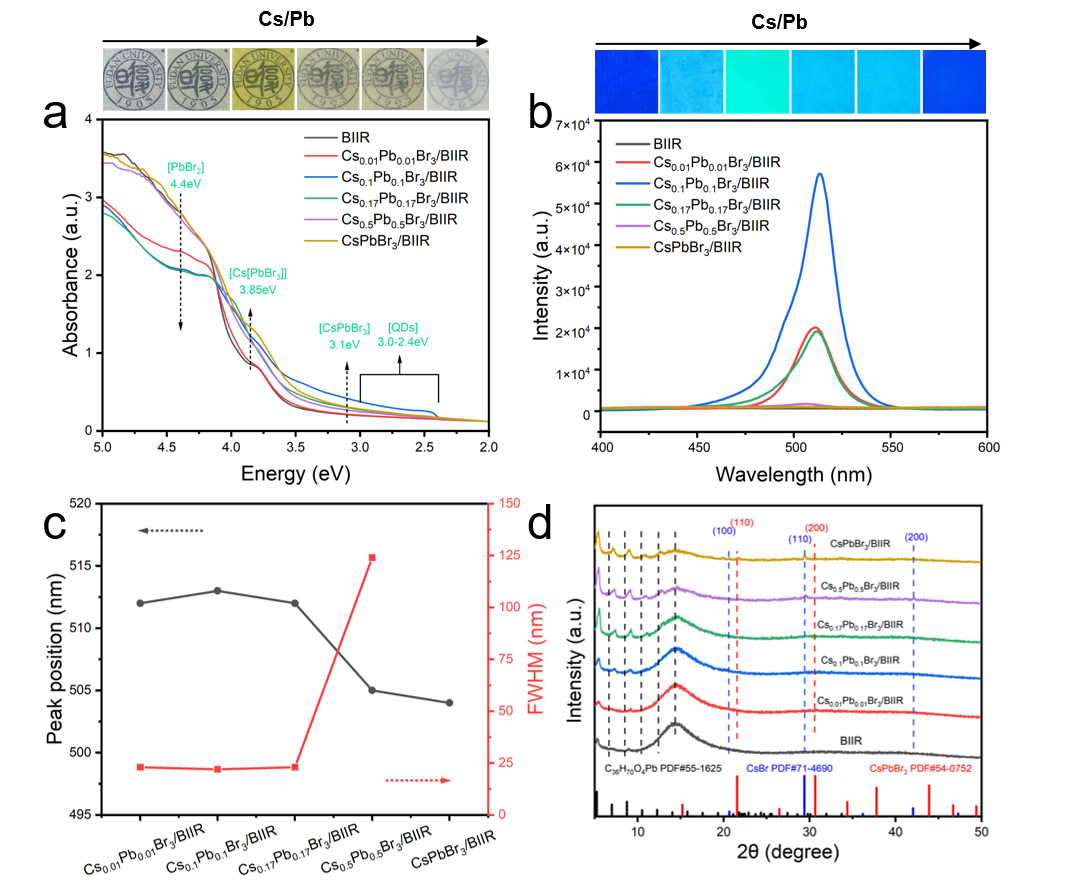


**Figure S1.** Absorption spectra (a), PL spectra (b), the maximum fluorescence peak and FWHM (c) , and XRD patterns (d) of CsPbBr_3_/BIIR composite films under different bromine contents.

As shown in Figure S1a, the characteristic peak at 3.79 eV in the BIIR curve corresponds to the π→π* electronic transition of the conjugated diene structure (-C=C-C=C-). With the addition of cesium and lead sources, when the Br/Cs molar ratio is greater than 18, the characteristic peak at 3.79 eV gradually disappears. Simultaneously, the perovskite exciton peak appears at 2.45 eV and gradually intensifies. Upon further increasing the amount of Cs and Pb sources, the perovskite exciton peak disappears, and the characteristic peak of BIIR reappears. The corresponding PL spectra of the CsPbBr_3_/BIIR composite under different bromine contents are shown in Figure S1b. When the Br/Cs molar ratio is greater than 18, the composite exhibits a perovskite PL peak at 515 nm, with a FWHM of 22 nm, indicating the rapid nucleation of pure cubic-phase CsPbBr_3_ nanocrystals under open-mixing shear force in a bromine-rich system. Further increasing the amounts of cesium source and lead source, the PL intensity of the composite gradually decreases and its position shifts to 505 nm, with an increase in the FWHM (Figure S1c). When the Br/Cs molar ratio reaches 3, the composite film basically does not emit light. Based on these experimental results, we can infer that bromine content plays a dominant role in the nucleation process. The ion reaction process of the precursor converting to perovskite quantum dots can be regulated by the addition of Cs and Pb sources to achieve a reversible process.

To study the influence of bromine content on the nucleation and growth of perovskite quantum dots (PQDs), different amounts of cesium and lead precursors were added to the equal amounts of brominated butyl rubber (BIIR). The mixed composite films were subjected to XRD crystal structure analysis. As shown in Figure S1d, the XRD pattern of the CsPbBr_3_/BIIR composite film exhibits a broad amorphous diffraction band at 10-20°, corresponding to the BIIR polymer, without displaying the PQD characteristic peaks of quantum dots. This indicates that the PQDs were effectively encapsulated by the polymer matrix. When the molar ratio of Cs:Pb:Br increases to 0.5:0.5:3, due to insufficient bromide ion concentration in the system, diffraction peaks corresponding to the reaction intermediate CsBr (PDF#71-4690) appear at 20.6° (100), 29.5° (110), and 42° (200). This is likely generated by a nucleophilic substitution reaction between the allylic bromine on the BIIR molecular chains and the cesium source. With further increase in lead and cesium precursors, diffraction peaks for cubic CsPbBr_3_ (PDF#54-0752) appear at 21.6° and 42.1°, corresponding to the (110) and (200) planes, respectively.

**1.2 Reaction Mechanism of CsPbBr_3_/BIIR composite films Under Different Thermodynamic Conditions**


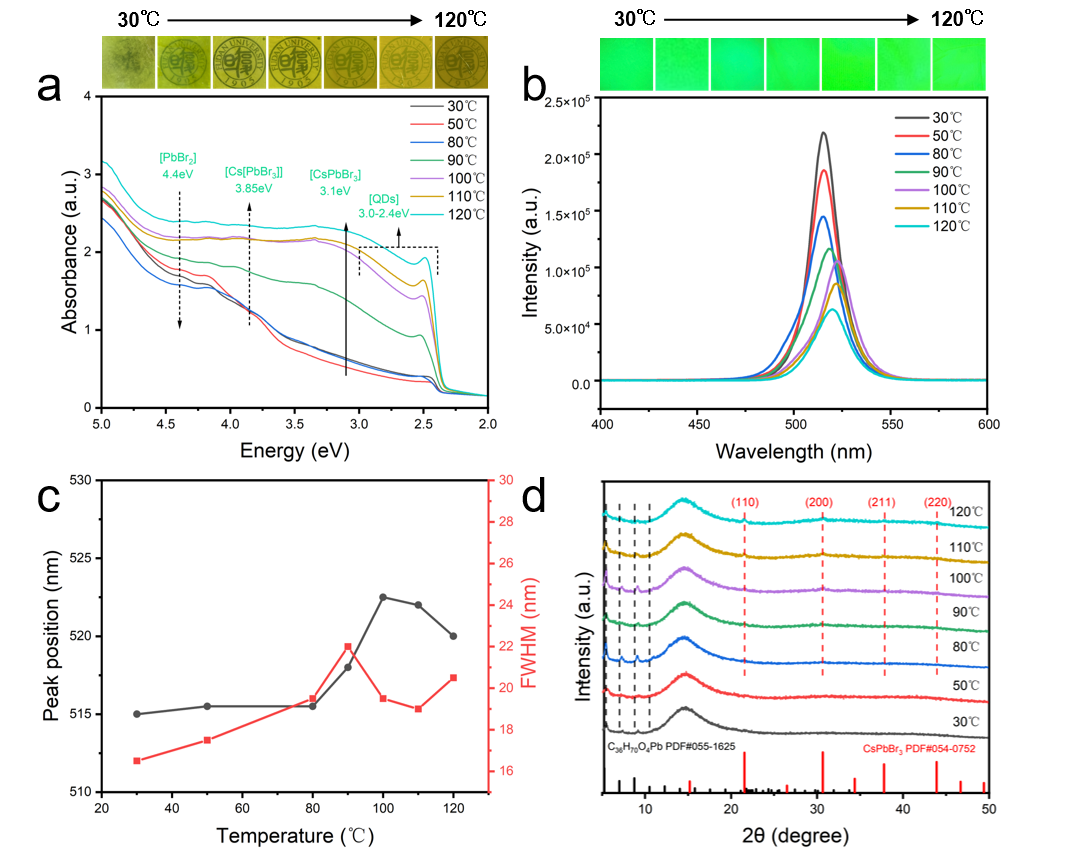


**Figure S2.** Absorption spectra (a), PL spectra (b), and the maximum fluorescence peak and FWHM (c) of Cs_0.1_Pb_0.1_Br_3_/BIIR composite films vulcanized at different temperature, and XRD spectra (d) of Cs_0.1_Pb_0.1_Br_3_/BIIR composite films under different sulfidation temperature.

Reaction temperature is a key factor affecting the thermodynamics growth of perovskite materials. To verify the effect of hot-pressing temperatures on the samples, the composite with Cs/Pb/Br molar ratio 0.1:0.1:3 was vulcanized at the temperature range of 30-120°C, and their luminescence properties were characterized. As shown in Figure S2a, the absorption spectrum of the film at 30°C shows a sharp excitonic peak at 2.45 eV, with no sharp peak at 3.91 eV. The corresponding PL peak is at 515 nm, with a FWHM of 17 nm, indicating that only pure cubic-phase CsPbBr_3_ nanocrystals are generated by open-mixing shear. When the vulcanization temperature reaches 90°C, we observe a sharp peak at 3.91 eV, and the first excitonic absorption peak at 2.45 eV shifts to 2.52 eV and its intensity increases significantly. This indicates that the composite film underwent further thermodynamic reactions at the vulcanization temperature of 90°C, generating cubic-phase CsPbBr_3_ and Cs_4_PbBr_6_ nanocrystals.

As shown in Figure S2b, with increasing temperature from 30°C to 80°C, the fluorescence intensity of the corresponding films continuously decreases, while their FWHM increases. This is primarily attributed to the non-localized and random distribution of lateral stress at different positions under the 15 T platen pressure applied during vulcanization, which promotes lateral growth of perovskite QDs along this direction and leads to the variation of grain size. When the vulcanization temperature rises to 90°C, the PL peak position of the composite film red-shifts from 515 nm to 520 nm, accompanied by an increase of FWHM (Figure S2c). This indicates that at this temperature, the lead stearate may further dissolve, resulting in more Pb^2+^ participating in the reaction and further generating quantum dots. With a continued increase in the vulcanization temperature, the fluorescence intensity of the composite film progressively decreases. The peak position red-shifts to a certain extent and then blue-shift, while the FWHM first decreases and then increases. This suggests that the excessive temperature leads to thermal quenching and thermal decomposition of the quantum dots.

To further investigate the the influence of sulfidation temperature on the crystal phase of CsPbBr_3_/BIIR composite films, the composite film with a Cs/Pb/Br molar ratio of 0.1:0.1:3 was selected as the research object. After 10 min of mixing, it was transferred to a flat vulcanizing machine and sulfided at different temperatures for 5 min. The resulting composite film was then subjected to the XRD test. As shown in Figure S2d, at the room temperature condition (30℃), the composite film curve exhibits residual lead stearate diffraction peaks at 5.4°, 7.4°, 9.2°, and 10.9°. Due to the strong interaction between lead stearate and the allyl bromide on the rubber molecular chains, all diffraction peaks shift toward larger angles. As the temperature increases from 30℃ to 80℃, due to the enhanced transparency of the CsPbBr_3_/BIIR composite film , the intensity of lead stearate diffraction peaks significantly increases. When the temperature reaches 90℃, the lead stearate further dissolves and undergoes a nucleophilic substitution reaction with the cesium source and bromine in the BIIR matrix, further generating perovskite quantum dots. At 21.6° (110) and 30.6° (200), pure cubic-phase CsPbBr_3_ diffraction peaks appear. When the temperature was raised to 120°C, distinct diffraction peaks at 21.6°, 30.6°, 37.9°, and 44° were observed, corresponding to the (110), (200), (211), and (220) planes of pure cubic-phase CsPbBr_3_ (PDF#54-0752), respectively. Meanwhile, the intensity of the lead stearate diffraction peaks gradually decreases.


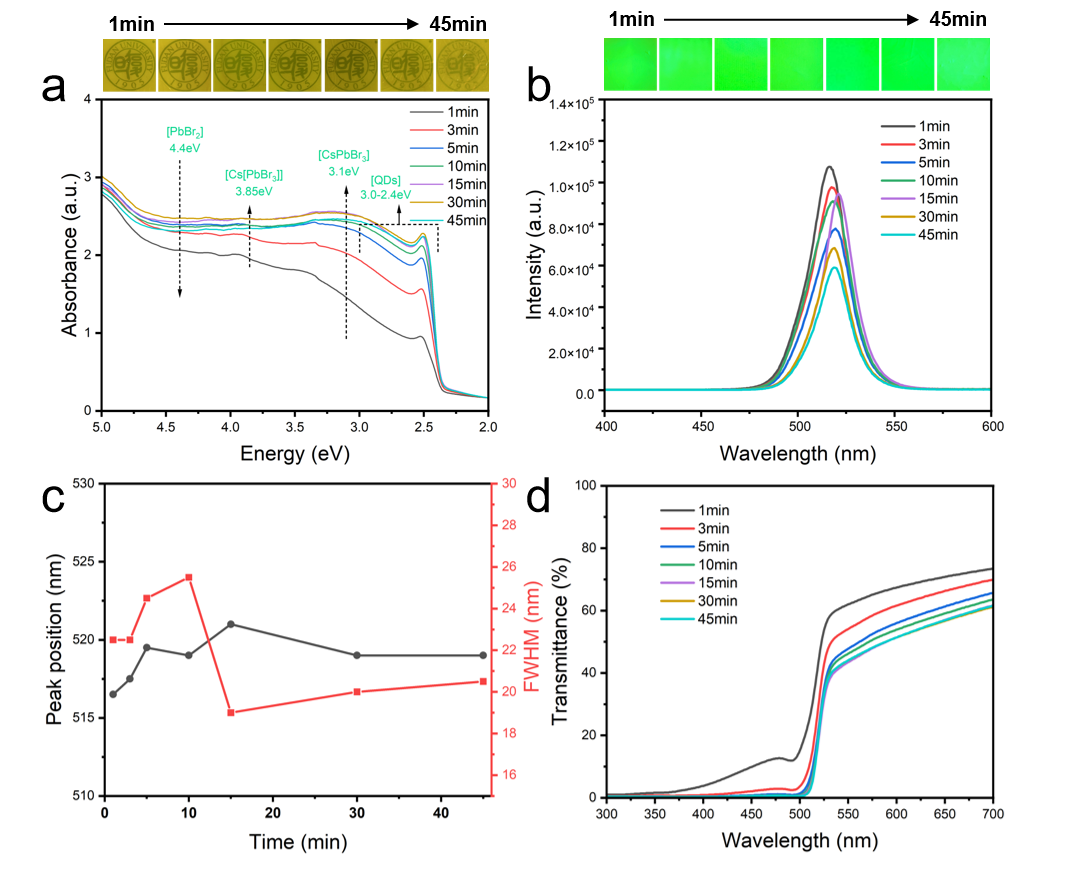


**Figure S3.** Absorption spectra (a), PL spectra (b), and the maximum fluorescence peak and FWHM (c) , and Transmission spectra (d) of CsPbBr_3_/BIIR composite films vulcanized at 100°C for different durationsCsPbBr_3_/BIIR composite films.

To reveal the evolution of the perovskite composite film under the influence of thermodynamics, the optical performance changes of the film at different times under vulcanization temperature of 100°C were observed (Figure S3). When vulcanized for 1 min, the first excitonic peak of the composite film shifts to 2.52 eV, and a sharp peak is observed at 3.91 eV. This indicates that the composite film underwent further thermodynamic reactions at the vulcanization temperature of 100°C, generating cubic-phase CsPbBr_3_ and Cs_4_PbBr_6_ nanocrystals. The first excitonic peak intensity of the composite film continuously increases with prolonged reaction time. When the vulcanization time reaches 15 min, the intensity of the first excitonic peak remains basically unchanged, and the sharp peak at 3.91 eV gradually disappears, indicating that with sufficient reaction time, the Cs_4_PbBr_6_ nanocrystal transforms into cubic-phase CsPbBr_3_ (Figure S3a). Further observing the changes in their PL spectra, as shown in Figure S3b, with prolonged vulcanization time, the PL peak of the composite film red-shifts from 516.5 nm to 521 nm, and the PL intensity first decreases and then increases. This is because the residual precursors at the vulcanization temperature of 100°C undergo further the thermodynamic reactions, causing the quantum dots to grow further. Meanwhile, the thermal quenching effect at this temperature leads to a decrease in the brightness of the composite film and the decomposition of a small amount of quantum dots. When the reaction time is 15 min, the peak position shifts to 519 nm, with a FWHM of 19 nm, which is the optimal reaction condition.

Further testing the transmittance change of the CsPbBr_3_/BIIR composite film with reaction time, as shown in Figure S3d. The transparency of composite films continuously decreases with prolonged reaction time. When the reaction time is 15 min, the transparency remains basically unchanged. This is because the lead source in the composite film further dissolves, leading to more Pb^2+^ participating in the ionic reaction, generating more quantum dots and causing a decrease in the transmittance of the composite film. To exclude the interference from the rubber, we tested the absorption spectra of BIIR at 100°C for different vulcanization times, as shown in Figure S4. The characteristic peak of the rubber at 3.79 eV, corresponding to the π→π* electronic transition of the conjugated diene structure (-C=C-C=C-) in the molecular chains, remains basically unchanged with prolonged vulcanization time.


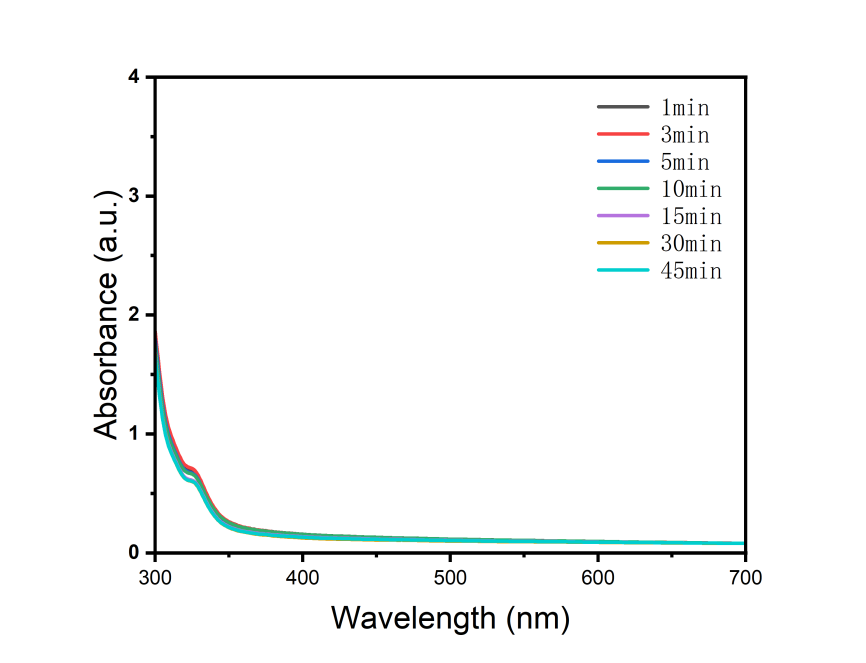


**Figure S4.** Absorption spectra of pure BIIR vulcanized at 100°C for different time.


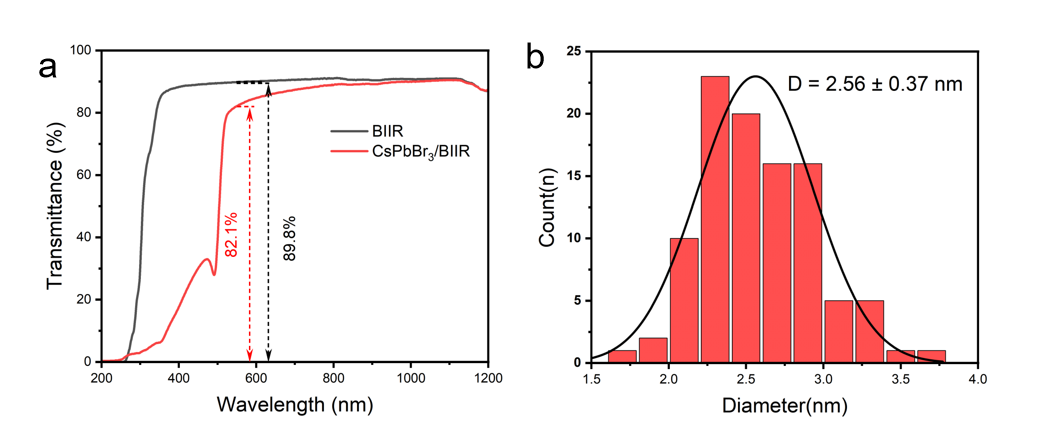


**Figure S5.** (a) Transmission spectra of pure BIIR and CsPbBr_3_/BIIR composite films with a thickness of 0.3 mm. (b) Particle size distribution histogram of quantum dots within the CsPbBr_3_/BIIR composite film.


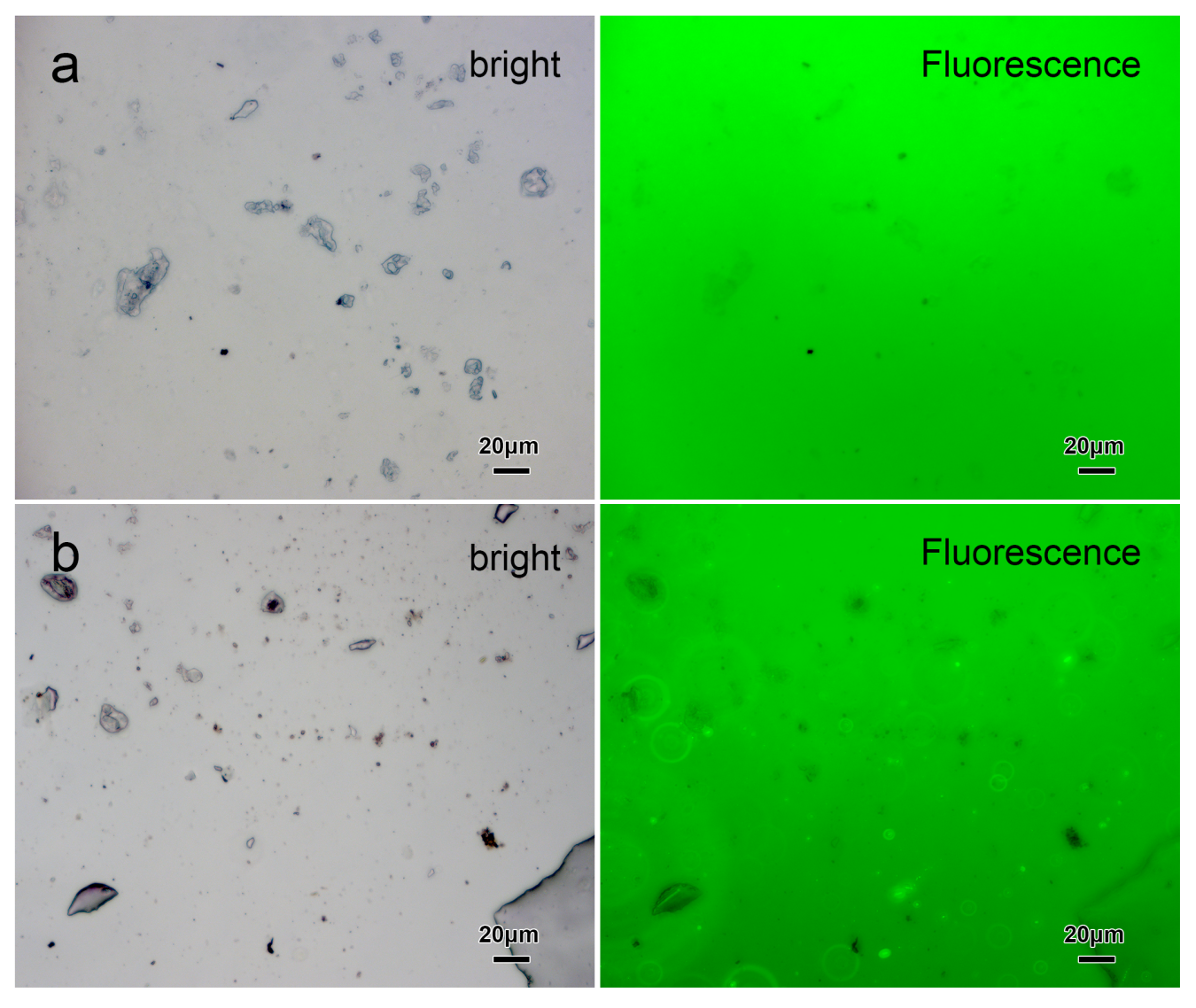


**Figure S6.** (a) Fluorescence microscope images of CsPbBr_3_/BIIR and CsPbBr_3_/IIR composite films.


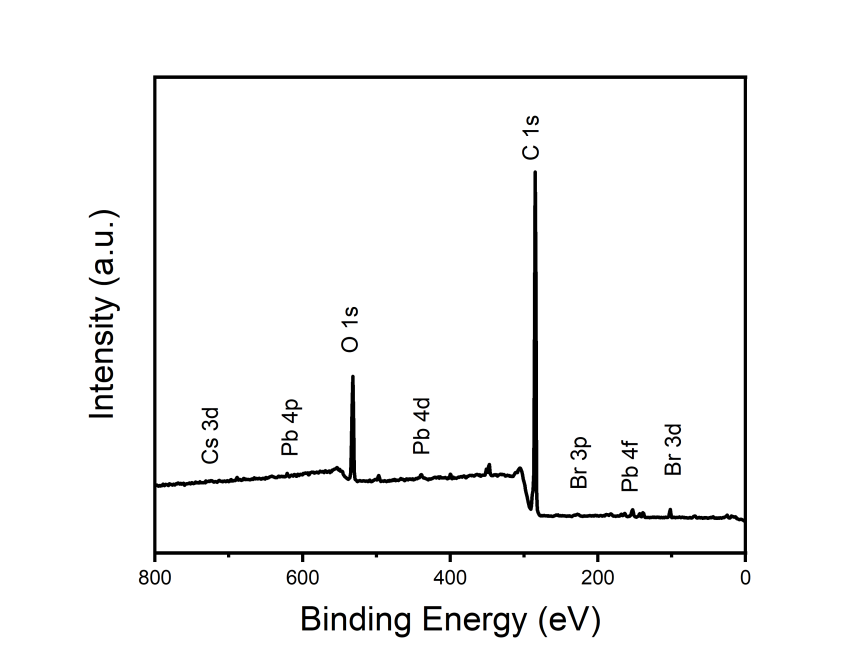


**Figure S7.** Full XPS survey scan of CsPbBr_3_/BIIR composite film.

**Table S1.** Elemental content of CsPbBr_3_/BIIR composite films with different bromine contents.

| Sample | O | Br | Cs | Au | Pb | C |
| --- | --- | --- | --- | --- | --- | --- |
| BIIR | 7.2 | 4.4 | 0.0 | 13.0 | 0.0 | 75.4 |
| Cs_0.01_Pb_0.01_Br_3_/BIIR | 6.4 | 4.1 | 0.0 | 12.3 | 0.1 | 77.0 |
| Cs_0.1_Pb_0.1_Br_3_/BIIR | 8.4 | 3.9 | 0.2 | 12.9 | 0.5 | 74.1 |
| Cs_0.17_Pb_0.17_Br_3_/BIIR | 6.9 | 4.3 | 0.3 | 15.9 | 0.6 | 71.9 |
| Cs_0.5_Pb_0.5_Br_3_/BIIR | 5.3 | 4.8 | 0.8 | 16.2 | 2.3 | 70.5 |
| CsPbBr_3_/BIIR | 6.8 | 4.0 | 1.5 | 13.3 | 3.4 | 71.0 |


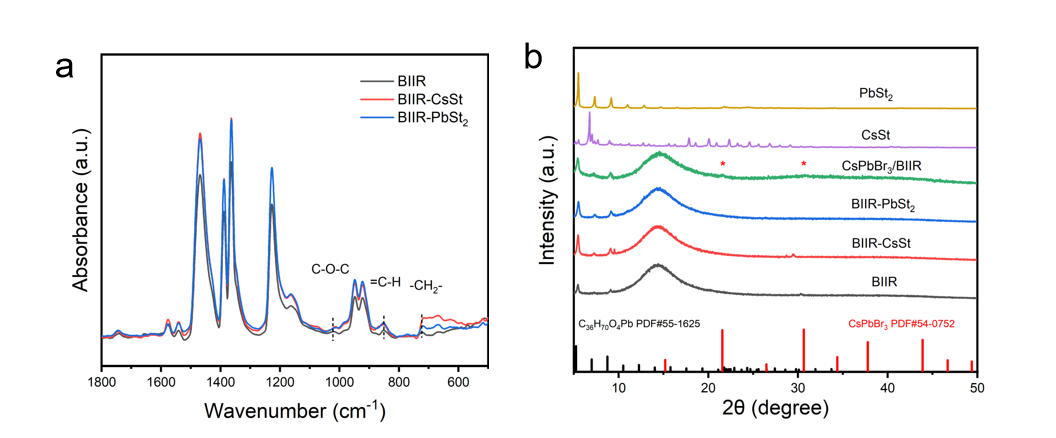


**Figure S8.** FTIR spectra (a) and XRD patterns (b) of BIIR mixed with Cs-source and Pb-source separately.

$$\text{I}\text{(}\text{t}\text{)=}\text{A}_{\text{1}}\text{e}^{\text{-}\frac{\text{t}}{\text{τ}_{\text{1}}}}\text{+}\text{A}_{\text{2}}\text{e}^{\text{-}\frac{\text{t}}{\text{τ}_{\text{2}}}}\text{+}\text{A}_{\text{3}}\text{e}^{\text{-}\frac{\text{t}}{\text{τ}_{\text{3}}}}\cdots\cdots\cdots\cdots\cdots\cdots\cdots\cdots\cdots\cdots\cdots\cdots\cdots\cdots\cdots\cdots(S1)$$

$$\text{τ}_{\text{avg}}\text{=}\frac{\text{A}_{\text{1}}\text{τ}_{\text{1}}^{\text{2}}\text{+}\text{A}_{\text{2}}\text{τ}_{\text{2}}^{\text{2}}\text{+}\text{A}_{\text{2}}\text{τ}_{\text{2}}^{\text{2}}}{\text{A}_{\text{1}}\text{τ}_{\text{1}}\text{+}\text{A}_{\text{2}}\text{τ}_{\text{2}}\text{+}\text{A}_{\text{2}}\text{τ}_{\text{2}}}\cdots\cdots\cdots\cdots\cdots\cdots\cdots\cdots\cdots\cdots\cdots\cdots\cdots\cdots\cdots(S2)$$

Where the short fluorescence lifetime *τ*₁ is the fast decay component, mainly related to defect-mediated non-radiative recombination; *τ*₂ is the intermediate decay component, related to exciton radiative recombination; And the slow decay component τ₃ is mainly related to Auger recombination. *A*ᵢ are the amplitudes of the corresponding components. *A*_1_, *A*_2_ and *A*_3_ represent the proportions of the short lifetime *τ*₁, medium lifetime *τ*₂, and long lifetime *τ*₃ at t = 0, respectively.

**Table S2.** Tri-exponential function fitting results of the PL decay curves for CsPbBr_3_/BIIR and CsPbBr_3_/IIR composite films.

| Sample | *A*_1_/% | *τ*_1_/ns | *A*_2_/% | *τ*_2_/ns | *A*_3_/% | *τ*_3_/ns | χ^2^ | *τ*_avg_/ns |
| --- | --- | --- | --- | --- | --- | --- | --- | --- |
| CsPbBr_3_/BIIR | 51.94 | 21.59 | 33.35 | 151.60 | 11.81 | 1548.32 | 0.9969 | 1189.73 |
| CsPbBr_3_/IIR | 20.35 | 1.42 | 64.56 | 11.91 | 14.37 | 45.47 | 0.9989 | 26.81 |


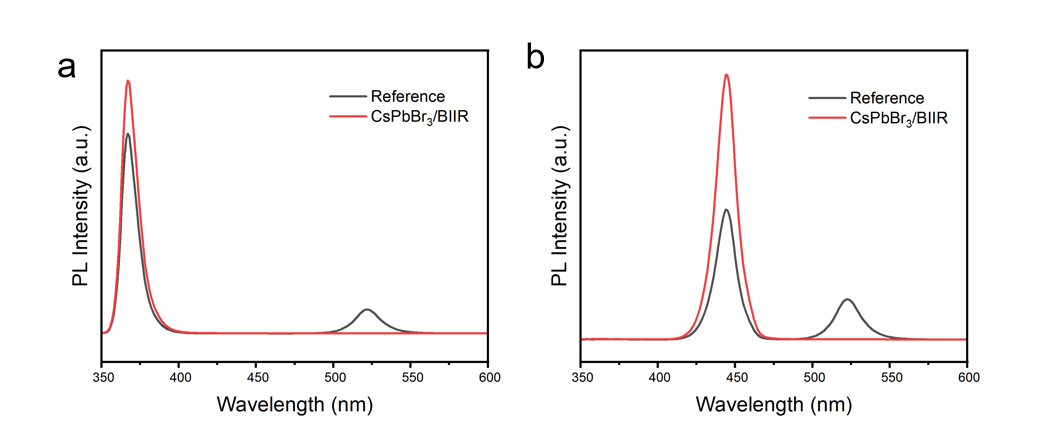


**Figure S9.** Typical PLQY of CsPbBr_3_/BIIR composite film under UV light (λ_d_ = 365 nm) (a) and blue light (λ_d_ = 450 nm) (b) excitation.


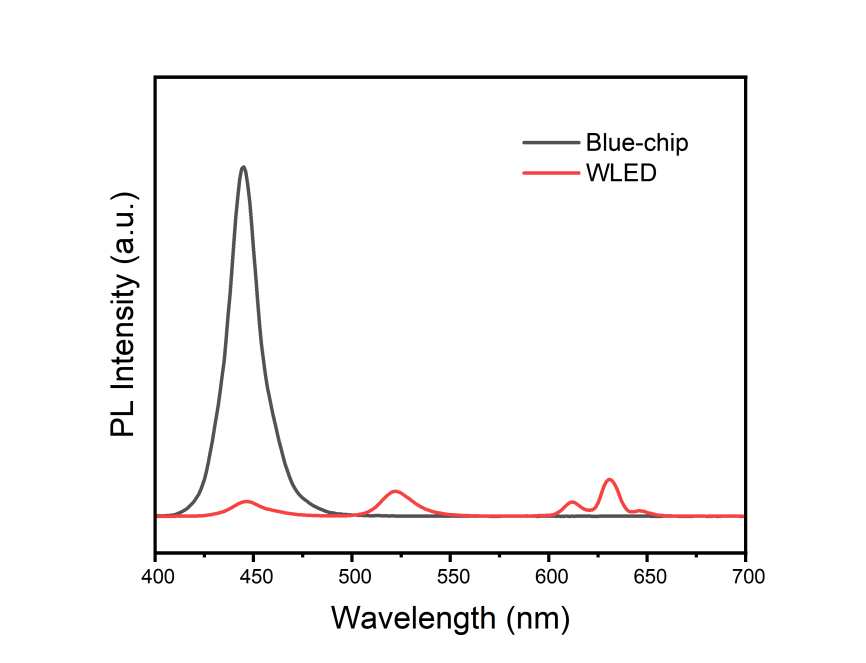


**Figure S10.** PL spectra of blue chip and the WLED device at a drive current of 1 mA.


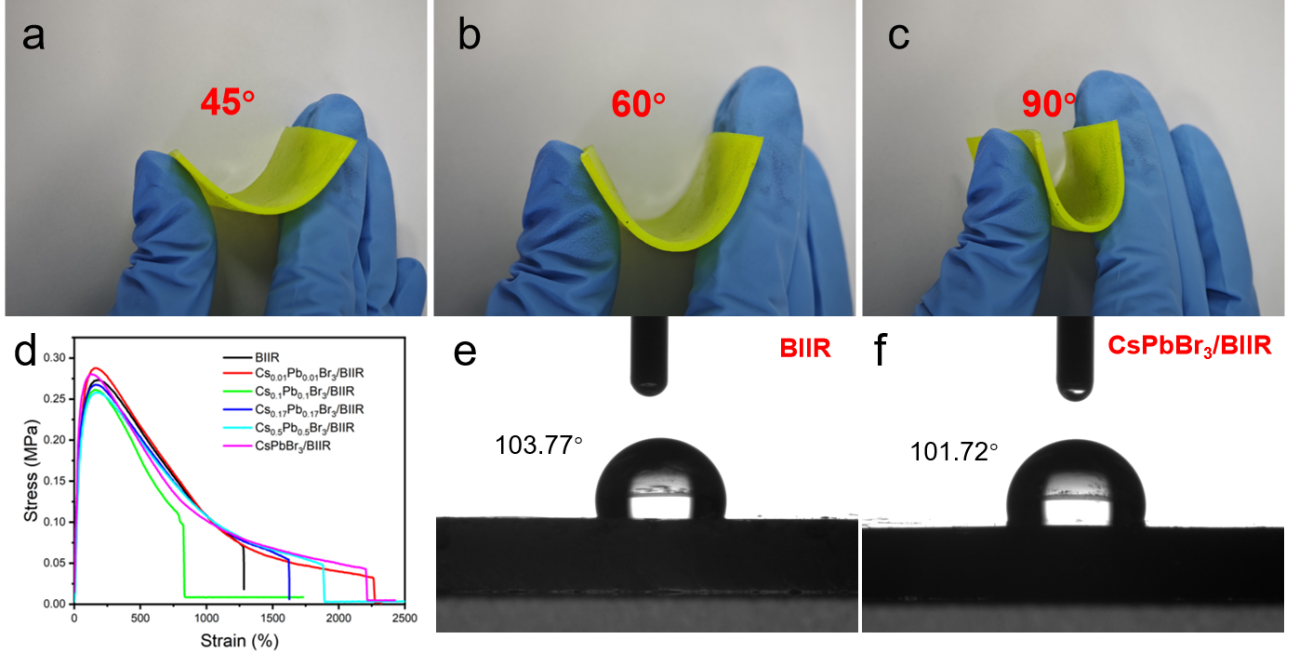


**Figure S11.** Optical photographs of the CsPbBr_3_/BIIR composite film under different bending angles of 40°, 60°, 90°(a-c). (d) Stress-strain curve of the CsPbBr_3_/BIIR composite films. Contact angle test of CsPbBr_3_/BIIR composite (e) and BIIR (f).


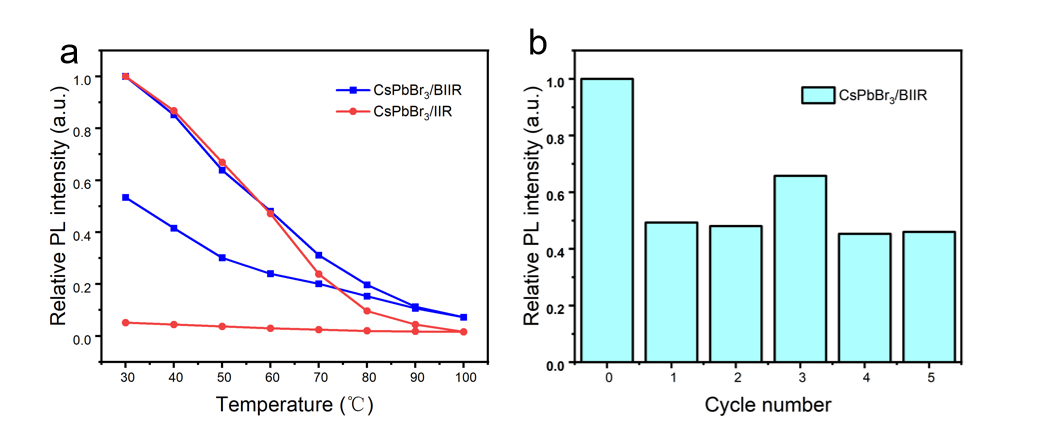


**Figure S12.** (a) Luminescence performance variation with temperature during a single thermal cycle (30-100°C) for CsPbBr_3_/BIIR and CsPbBr_3_/IIR composite films. (b) Variation of initial luminescence performance of the CsPbBr_3_/BIIR composite film after multiple heating cycles to 100°C.

**Table S3.** Summary of optical performance and stability of perovskite-organic polymer composite

| Sample | Emission center | FWHM | Color gamut | Chromatic coordinate | CCT | Storage@R.T. stability | Water stability | Ref. |
| --- | --- | --- | --- | --- | --- | --- | --- | --- |
| CsPbBr_3_/BIIR | 523 nm | 19 nm | 132% | (0.333, 0.338) | 5200 K | 90% (30 days) | 99.8% (30 days) | This work |
| OM-CsPbBr_3_/PS | 522 nm | 23 nm | N/A | (0.33, 0.32) | N/A | N/A | ≈100% (10 days) | [1] |
| CsPbBr_3_-PCA | 514 nm | 20 nm | 127.3% | (0.311, 0.420) | 6142 K | N/A | ≈100% (30 months) | [2] |
| CsPbBr_3_@HPβCD@PFOS | 519 nm | 17 nm | N/A | (0.316, 0.318) | 6388 K | 50% (591 days) | >85% (136 days) | [3] |
| CsPbBr_3_/PMMA | 520 nm | 19 nm | 126.5% | (0.336, 0.342) | 5318 K | N/A | ≈53% (15 days) | [4] |
| CsPbBr_3_/PS | N/A | 20 nm | 128%@NTSC1953 | (0.333, 0.322) | N/A | N/A | N/A | [5] |
| FAPbBr_3_/PMMA | 534 nm | 21 nm | N/A | (0.37, 0.35) | 4000 K | N/A | ≈90% ( 45 days) | [6] |
| CsPbBr_3_@PDPEP-*co*-S | 522 nm | N/A | N/A | (0.31, 0.32) | N/A | N/A | 33% (30 days) | [7] |
| CsPbBr_3_/PMSQ AG | 520 nm | N/A | N/A | (0.386, 0.333) | 3444 K | 71% (14 days) | 50% (14 days) | [8] |
| SiO_2_@CsPbBr_3_@(PS/L) APNCCs | 524 nm | N/A | 120% | (0.334, 0.332) | N/A | 96.1% (60 days) | 81.8% (24 h) | [9] |

**References:**

[1] W. Fan, S. Wang, C. Gong, Z. Feng, B. Li, Z. Yang, J. Song, "Synthesis of Highly Efficient and Dispersible OM-CsPbBr_3_ Quantum Dots Integrated into Polymers by Surface Medium Anchoring Strategy," *Chem. Eng. J.* **2025**, *520*, 165951.

[2] H. Jin, G. Yeong Park, M. Kyong Kim, J. Cha, D. Seok Ham, M. Kim, "Eco-Friendly Solvent-Processible and Highly Luminescent Perovskite Nanocrystals with Polymer Zwitterions for Air-Stable Optoelectronics," *Chem. Eng. J.* **2023**, *459*, 141531.

[3] T. Tian, M. Yang, Y. Fang, S. Zhang, Y. Chen, L. Wang, W.-Q. Wu, "Large-Area Waterproof and Durable Perovskite Luminescent Textiles," *Nat. Commun.* **2023**, *14*, 234.

[4] Z. Wang, R. Fu, F. Li, H. Xie, P. He, Q. Sha, Z. Tang, N. Wang, H. Zhong, "One‐Step Polymeric Melt Encapsulation Method to Prepare CsPbBr_3_ Perovskite Quantum Dots/Polymethyl Methacrylate Composite with High Performance," *Adv. Funct. Mater.* **2021**, *31*, 2010009.

[5] W. Fan, S. Wang, Z. Yang, J. Yao, L. Xu, J. Song, "In Situ Formation of Luminescent Perovskite Quantum Dot/Polymer Composites: Scalable Synthesis, Continuous Processing and Functional Applications," *Adv. Mater.* **2025**, *37*, 2505600.

[6] H. Zhu, M. Cheng, J. Li, S. Yang, X. Tao, Y. Yu, Y. Jiang, "Independent Dispersed and Highly Water-Oxygen Environment Stable FAPbBr_3_ QDs-Polymer Composite for Down-Conversion Display Films," *Chem. Eng. J.* **2022**, *428*, 130974.

[7] W. Yang, L. Fei, F. Gao, W. Liu, H. Xu, L. Yang, Y. Liu, "Thermal Polymerization Synthesis of CsPbBr_3_ Perovskite-Quantum-Dots@Copolymer Composite: Towards Long-Term Stability and Optical Phosphor Application," *Chem. Eng. J.* **2020**, *387*, 124180.

[8] Y.-T. Hsieh, Y.-F. Lin, W.-R. Liu, "Enhancing the Water Resistance and Stability of CsPbBr_3_ Perovskite Quantum Dots for Light-Emitting-Diode Applications through Encapsulation in Waterproof Polymethylsilsesquioxane Aerogels," *ACS Appl. Mater. Interfaces* **2020**, *12*, 58049.

[9] J. Wang, M. Zhang, Y. Liu, Y. a. Huang, Y. Zhang, J. Jiang, H. Li, J. Chen, Z. Lin, "Ultrastable Highly-Emissive Amphiphilic Perovskite Nanocrystal Composites via the Synergy of Polymer-Grafted Silica Nanoreactor and Surface Ligand Engineering for White Light-Emitting Diode," *Nano Energy* **2022**, *98*, 107321.
